# Fe_3_O_4_@SiO_2_@SBA-3@CPTMS@Arg-Cu: preparation, characterization, and catalytic performance in the conversion of nitriles to amides and the synthesis of 5-substituted 1*H*-tetrazoles†

**DOI:** 10.1039/d3na00318c

**Published:** 2024-03-26

**Authors:** Zahra Heidarnezhad, Arash Ghorbani-Choghamarani, Zahra Taherinia

**Affiliations:** a Department of Chemistry, Faculty of Science, Ilam University Ilam Iran; b Department of Organic Chemistry, Faculty of Chemistry and Petroleum Sciences, Bu-Ali Sina University Hamedan 6517838683 Iran a.ghorbani@basu.ac.ir arashghch58@yahoo.com +98 8138380709 +98 8138282807

## Abstract

A novel, efficient, and recyclable mesoporous Fe_3_O_4_@SiO_2_@SBA-3@CPTMS@Arg-Cu nanocatalyst was synthesized by grafting l-arginine (with the ability to coordinate with Cu) onto a mixed phase of a magnetic mesoporous SBA-3 support. The catalyst was characterized using several techniques, including Fourier-transform infrared (FT-IR) spectroscopy, thermogravimetric analysis (TGA), vibrating sample magnetometry (VSM), X-ray diffraction (XRD) analysis, N_2_ adsorption–desorption analysis, scanning electron microscopy (SEM), X-ray photoelectron spectroscopy (XPS), energy dispersive X-ray (EDX) analysis, and atomic absorption spectroscopy (AAS). The resulting solid material possessed a surface area of 145 m^2^ g^−1^ and a total pore volume of 34 cm^3^ g^−1^. The prepared mesoporous material was studied as a practical, recyclable, and chemoselective catalyst in some organic functional group transformations such as the conversion of nitriles to amides and synthesis of 5-substituted 1*H*-tetrazoles. This novel magnetic nanocatalyst proved to be effective and provided the products in high to excellent yields under green solvent conditions. Meanwhile, the as-prepared Fe_3_O_4_@SiO_2_@SBA-3@CPTMS@Arg-Cu demonstrated excellent reusability and stability under reaction conditions, and its catalytic activity shown only a slight decrease after seven consecutive runs. Therefore, the as-synthesized magnetic Fe_3_O_4_@SiO_2_@SBA-3@CPTMS@Arg-Cu has broad prospects for practical applications, and offers various benefits such as simplicity, nontoxicity, low cost, simple work-up, and an environmentally benign nature.

## Abstract 2

A novel, efficient, and recyclable mesoporous Fe_3_O_4_@SiO_2_@SBA-3@CPTMS@Arg-Cu nanocatalyst was synthesized by grafting l-arginine (with the ability to coordinate with Cu) onto a mixed phase of a magnetic mesoporous SBA-3 support.

## Graphical abstract

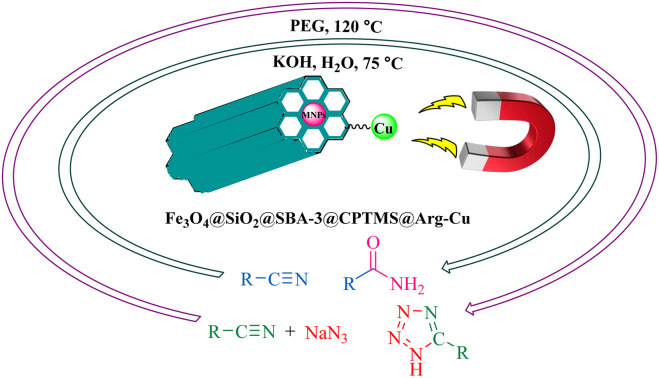

## 1. Introduction

**Scheme 1:**
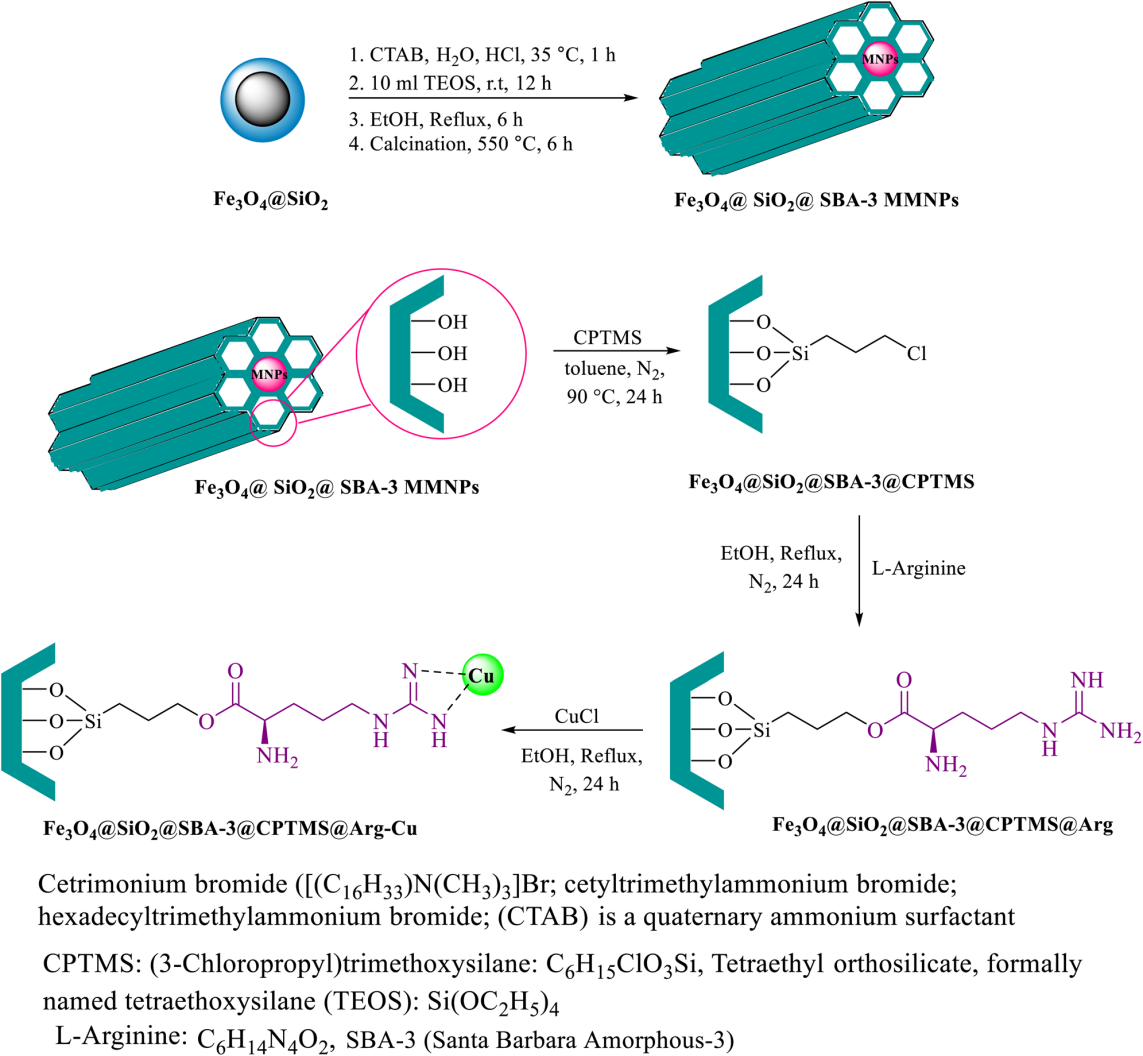
Preparation of Fe_3_O_4_@SiO_2_@SBA-3@CPTMS@Arg-Cu.

The existence of larger mesopores plays a key role in enhancing activity efficiency, as it leads to the formation of metals with larger sizes, resulting in less metal–support interaction, which is more favorable for reaction. In recent years, various types of mesoporous materials such as mobile crystalline materials (MCM-48, MCM 41, and MCM-50)^1^ and SBA-type materials (SBA-15, SBA-3)^2^ have been developed. These materials have found critical applications in various fields^3–9^ due to their unique properties such as high surface areas and well-ordered large pore systems. Among the SBA-type materials, several catalytic reactions using SBA-15 as a support for metal active phases have been investigated and showed promising results.^9–13^ Moreover, due to the contribution of some micropores connecting the mesopores, SBA-15 silica mesoporous materials with a more regular structure and thicker walls were shown to have much higher stability than MCM-41.^14^ SBA-3 (pore diameter > 3 nm) and SBA-1 (pore diameter > 2 nm) can contain some micropores too.^15^ However, the microporosity of SBA-3 is higher than that of SBA-15 (ref. 15) but with similar thermal and hydrothermal resistances.^15^ In the literature, applications of SBA-3 as a catalyst support are rare.^16,17^ However, special attention has been focused on the preparation of various magnetic nanostructures owing to their unique characteristics and potential applications in various fields.^18–23^ The immobilization of magnetic nanoparticles coated with mesoporous materials is of considerable technological importance from both economic and the environmental point of views. The separation of magnetic catalysts *via* magnetic separation is an attractive alternative to filtration or centrifugation for separating solid catalysts. Tetrazoles are a class of synthetic organic heterocyclic compounds that have numerous applications, especially in the fields of medicine,^24^ biochemistry,^25^ pharmacology,^26^ and in industry as materials,^27^*e.g.*, in photography,^28^ imaging chemicals,^29^ and the military.^30^ Various methods have been reported in the literature for the preparation of tetrazoles.^31^ A general and versatile procedure for the preparation of tetrazole is based on [3 + 2] cycloaddition of an azide ion and organic nitriles in the presence of a catalyst.^32–35^ However, the disadvantages of this method compared with other methods include the typically long reaction times, use of expensive and toxic reagents and high boiling solvents, harsh reaction conditions, low yield, and tedious work-up, which lead to unavoidable environmental and safety problems. In contrast, amides are an important key intermediate in many organic transformations that are widely applied in medicine,^36,37^ biochemistry,^38^ and materials science.^39^ Various approaches have been used to synthesize amides, such as condensation of carboxylic acid and esters with amines,^40^ reaction between alcohols/aldehydes with amines,^41^ and hydroamination of unsaturated hydrocarbons.^42^ Besides these methods, a practical method to form amides is the hydration of nitriles.^43^ Moreover, nitrile hydration reactions are atom-efficient and allow avoiding the generation of environmental waste. Based on these advantages, the hydration of nitriles to amides is a well-established method in the pharmaceutical industry for the synthesis of various amides for large-scale production. Various catalysts have been explored, including the commonly used strong acids and bases.^44,45^ However, the use of these acidic reagents in many cases may not be appropriate, particularly for compounds possessing acid-sensitive substrates. Herein, we introduce a facile synthesis approach for the preparation of Fe_3_O_4_@SiO_2_@SBA-3@CPTMS@Arg-Cu by grafting l-arginine (with the ability to coordinate with Cu) onto a mixed phase of a magnetic mesoporous SBA-3 support as a novel and magnetically recoverable heterogeneous catalyst. The green and environmentally sustainable nature of the prepared catalyst is a suitable factor for developing this composite material. One of the major advantages of the present study is that it demonstrates an eco-friendly and cost-effective method for the conversion of nitriles to amides and for the synthesis of 5-substituted 1*H*-tetrazoles. Furthermore, using a high surface area material has a synergistic effect that significantly enhances the catalyst performance. Moreover, the catalyst could be easily recovered by a facile separation process *via* magnetic forces and recycled several times without significant loss of its catalytic activity. Synthetic steps for the preparation of the catalyst are shown in Scheme 1.

## 2. Experimental

### 2.1. Preparation of Fe_3_O_4_@SiO_2_@SBA-3@CPTMS@Arg-Cu

Fe_3_O_4_@SiO_2_@SBA-3 was readily synthesized in a similar way to a previously reported work.^17^ Briefly, 1.00 g of the obtained Fe_3_O_4_@SiO_2_@SBA-3 powder was dispersed in a mixture of 25 mL of toluene using sonication for 30 min, and 1.5 mL of (3-chloropropyl)triethoxysilane (CPTES) was then added to the mixture. The reaction mixture was stirred at 90 °C for 24 h. Then, the prepared Fe_3_O_4_@SiO_2_@SBA-3@CPTMS was filtered, washed with ethanol (70% v/v), and dried at room temperature. The obtained Fe_3_O_4_@SiO_2_@SBA-3@CPTMS (1 g) was dispersed in ethanol (25 mL in 20 min), and then l-arginine (2 mmol) was added to the reaction mixture and stirred (24 h at 80 °C). Then, the resulting nanoparticles (Fe_3_O_4_@SiO_2_@SBA-3@CPTMS@Arg) were filtered, washed with ethanol (70% v/v), and dried at room temperature. The obtained Fe_3_O_4_@SiO_2_@SBA-3@CPTMS@Arg (0.5 g) was dispersed in 25 mL of ethanol (70% v/v) by sonication for 20 min, and then copper(i) chloride (2 mmol) was added to the reaction mixture. The reaction mixture was stirred at 80 °C for 24 h. Finally, a brown-colored product [Fe_3_O_4_@SiO_2_@SBA-3@CPTMS@Arg-Cu] was obtained after filtering, washing using ethanol (70% v/v), and drying at room temperature.

### 2.2. General procedure for the conversion of nitriles to amides

A mixture of nitrile (1 mmol), potassium hydroxide (2 mmol), and Fe_3_O_4_@SiO_2_@SBA-3@CPTMS@Arg-Cu (40 mg) in water was heated at 75 °C for an appropriate period. The reaction progress was monitored through TLC. After the completion of the reaction, the precipitate was extracted with ethyl acetate, washed with double-distilled water, dried over Na_2_SO_4_, concentrated under reduced pressure, and finally purified by column chromatography on silica gel using hexane : ethylacetate (4 : 1) to afford the desired product.

### 2.3. General procedure for the synthesis of 5-substituted 1*H*-tetrazoles

A mixture of nitrile (1 mmol), sodium azide (1.1 mmol), and Fe_3_O_4_@SiO_2_@SBA-3@CPTMS@Arg-Cu (40 mg) in polyethylene glycol was heated at 120 °C for an appropriate period. The reaction progress was monitored through TLC. After the completion of the reaction (monitored by TLC), the precipitate was extracted with ethyl acetate, washed with double-distilled water, dried over Na_2_SO_4_, concentrated under reduced pressure, and purified by column chromatography on silica gel using hexane : ethylacetate (4 : 1) to afford the desired product.

## 3. Results and discussion

**Fig. 1:**
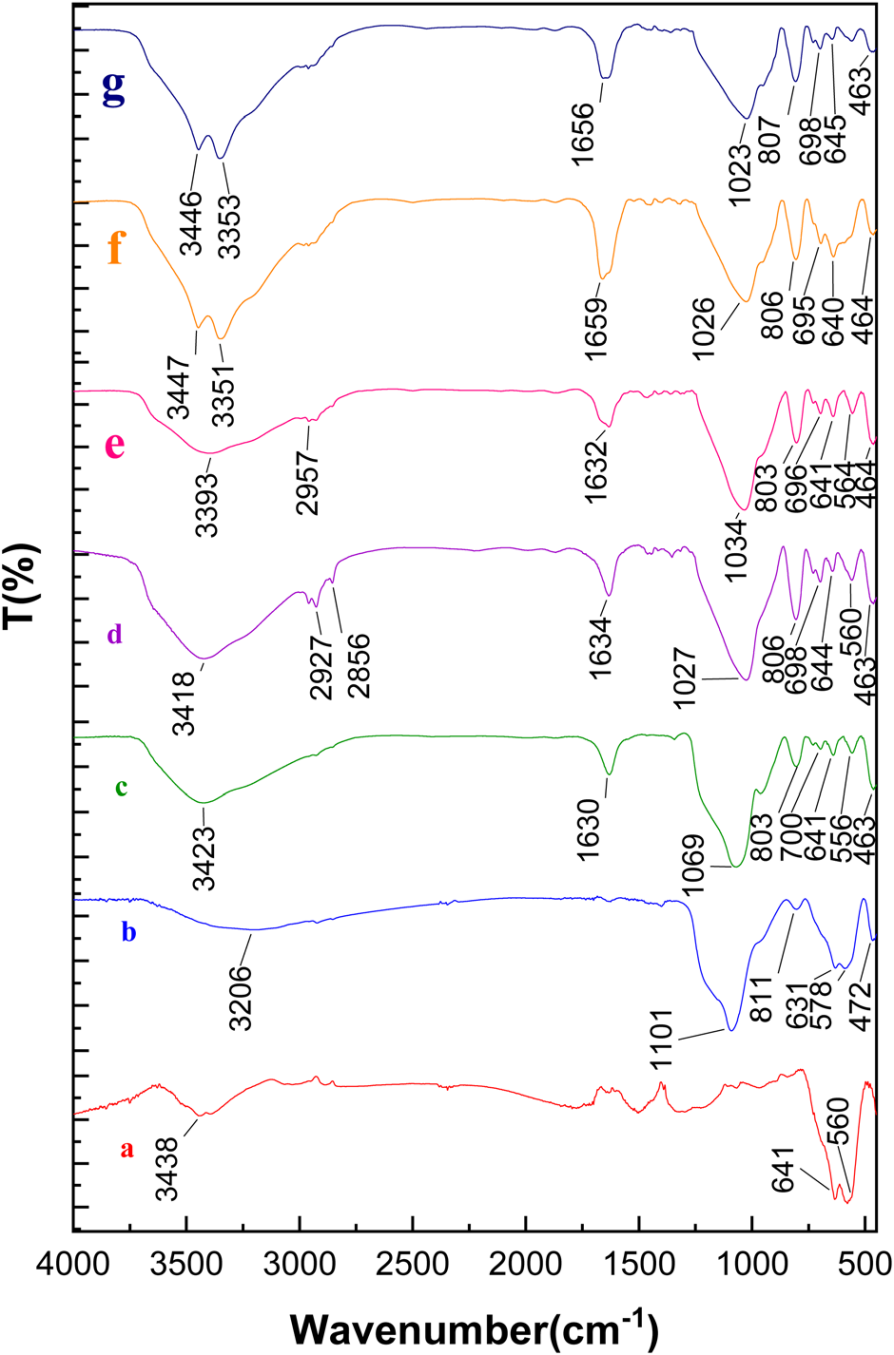
FT-IR spectra of (a) Fe_3_O_4_, (b) Fe_3_O_4_@SiO_2_, (c) Fe_3_O_4_@SiO_2_@SBA, (d) Fe_3_O_4_@SiO_2_@SBA-3@CPTMS, (e) Fe_3_O_4_@SiO_2_@SBA-3@CPTMS@Arg, (f) Fe_3_O_4_@SiO_2_@SBA-3@CPTMS@Arg-Cu (before recovery), and (g) Fe_3_O_4_@SiO_2_@SBA-3@CPTMS@Arg-Cu (after recovery).

After the preparation of the catalyst, the structure of Fe_3_O_4_@SBA-3@CPTMS@Arg-Cu was extensively studied using various techniques such as FT-IR spectroscopy, TGA, VSM, XRD, BET, SEM, XPS, EDX, and AAS analysis. Fig. 1 shows the FT-IR spectra of Fe_3_O_4_ (a), Fe_3_O_4_@SiO_2_ (b), Fe_3_O_4_@SiO_2_@SBA (c), Fe_3_O_4_@SiO_2_@SBA-3@CPTMS (d), Fe_3_O_4_@SiO_2_@SBA-3@CPTMS@Arg (e), and Fe_3_O_4_@SiO_2_@SBA-3@CPTMS@Arg-Cu (f). As can be seen from Fig. 1a, there was a peak at 3438 cm^−1^ belonging to the O–H bond of nanomagnetic mesoporous materials and bands at 560 and 641 cm^−1^ could be assigned to the Fe–O stretching absorption. For all the samples, the absorption bands at around 1081–1066 cm^−1^ and 800–806 cm^−1^ could be attributed to asymmetric and symmetric stretching vibrations of Si–O–Si, stretching vibration of Si–O–H, and bending vibration of Si–O–Si. FTIR spectra for Fe_3_O_4_@SiO_2_@SBA-3@CPTMS showed an absorption peak at 2926–2959 cm^−1^, which was assigned to the C–H stretching vibration modes of the methylene group (Fig. 1d). The FT-IR analysis of Fe_3_O_4_@SiO_2_@SBA-3@CPTMS@Arg showed a stretching vibration for the C=O bond at 1632 cm^−1^ (Fig. 1e). This verified that the l-arginine was anchored on the surface of the magnetite. The C=N stretching vibration could be identified in the FT-IR spectrum of the Fe_3_O_4_@SiO_2_@SBA-3@CPTMS@Arg-Cu, which appeared at a higher frequency compared to that of Fe_3_O_4_@SiO_2_@SBA-3@CPTMS@Arg (Fig. 1f).

**Fig. 2:**
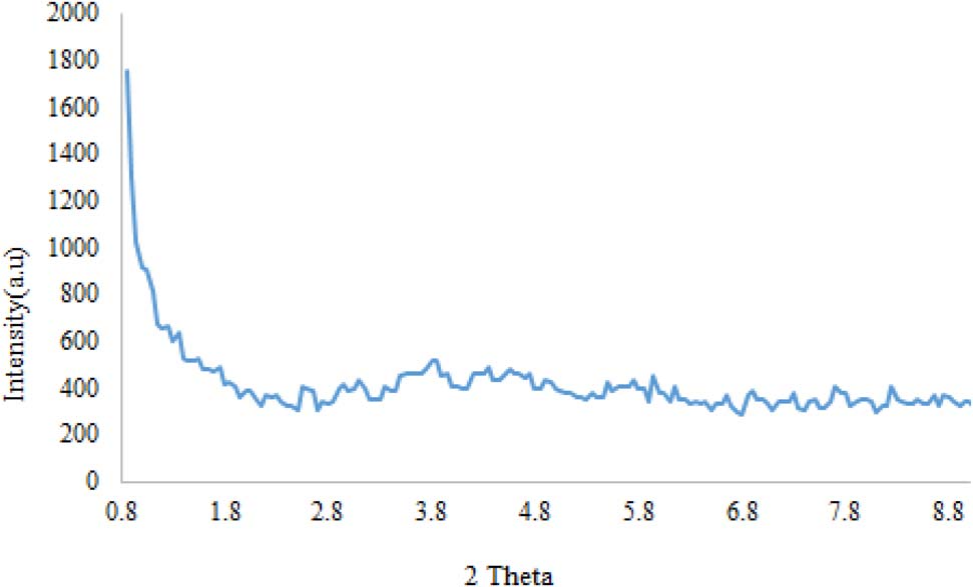
Low-angle XRD pattern of SBA-3.

**Fig. 3:**
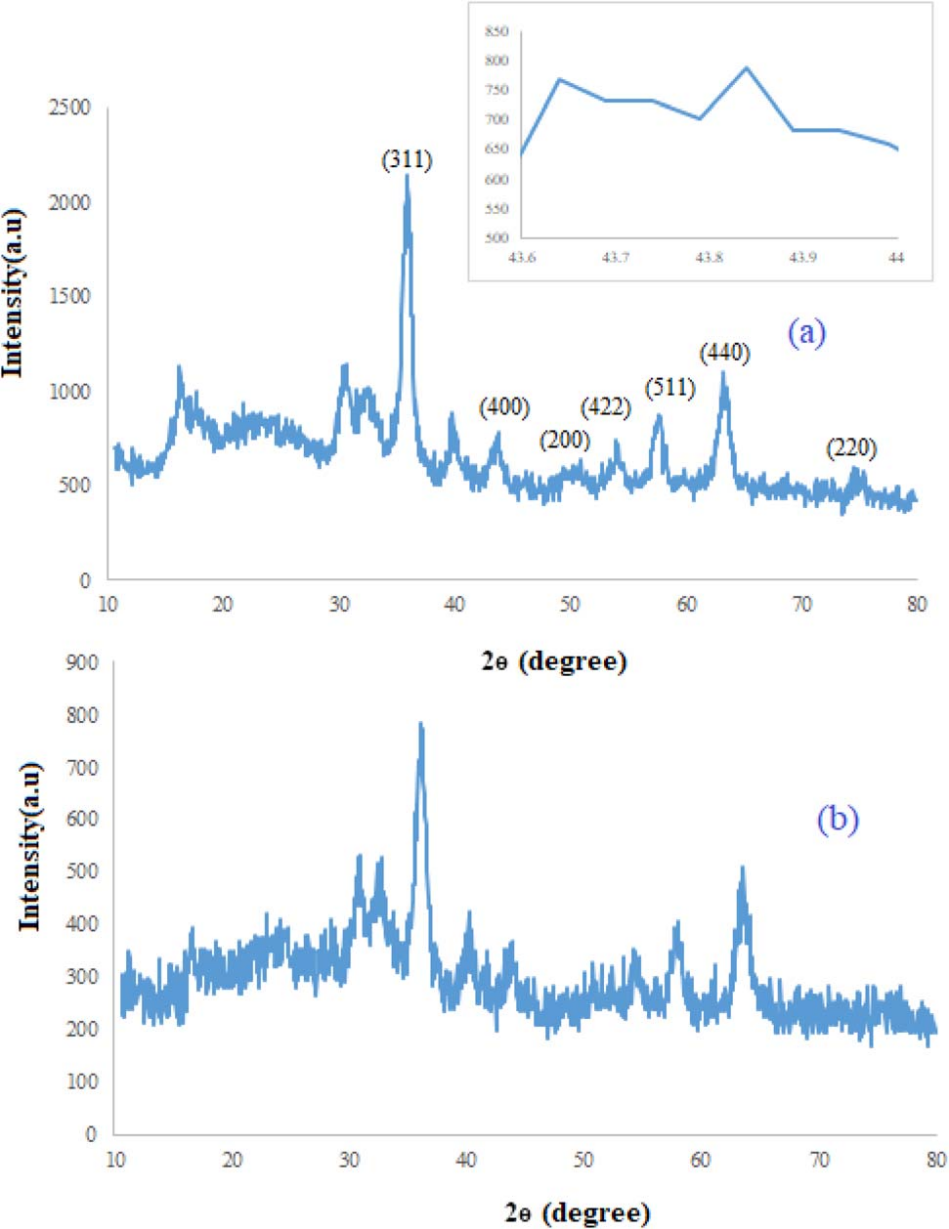
(a) XRD patterns of Fe_3_O_4_@SiO_2_@SBA-3@CPTMS@Arg-Cu (fresh catalyst) and (b) Fe_3_O_4_@SiO_2_@SBA-3@CPTMS@Arg-Cu after recovery.

The structure and phase purity of Fe_3_O_4_@SiO_2_@SBA-3@CPTMS@Arg-Cu and SBA-3 were studied using X-ray diffraction (XRD) analysis (Fig. 2 and 3). Fig. 2 shows typical peaks of a well-organized porous structured material with the main reflection in 2*θ* at 0.89° and other low-intensity reflections at 1.6° and 1.7°. In the low-angle XRD analysis, three diffraction planes were found, (100), (110), and (200), indicating a well-ordered hexagonal lattice.^46^

The wide-angle X-ray powder diffraction patterns of Fe_3_O_4_@SiO_2_@SBA-3@CPTMS@Arg-Cu are shown in Fig. 3. The XRD results showed diffraction peaks at 2*θ* of 35.7°, 43.6°, 53.85°, 57.31°, and 62.97°, which could be attributed to the (311), (400), (422), (511), and (440) reflections, respectively (JCPDS card no. 25–0283), which have been previously reported in the literature.^47^ This means that the multiple steps followed in the catalyst preparation did not change the crystal structure of the Fe_3_O_4_ cores. Besides, diffraction peaks of copper located at 43.7°, 50.7°, and 74.3° were observed, corresponding to the (111), (200), and (220) planes of the fcc structure, respectively (JCPDS 04-0836) (Fig. 3).^48,49^ In addition, the broad peak around 2*θ* = 15°–30° was attributed to the amorphous SiO_2_ layer around Fe_3_O_4_ as a shell of the core and proved the formation of the Fe_3_O_4_@SiO_2_ magnetic nanocomposite.^50,51^

**Fig. 4:**
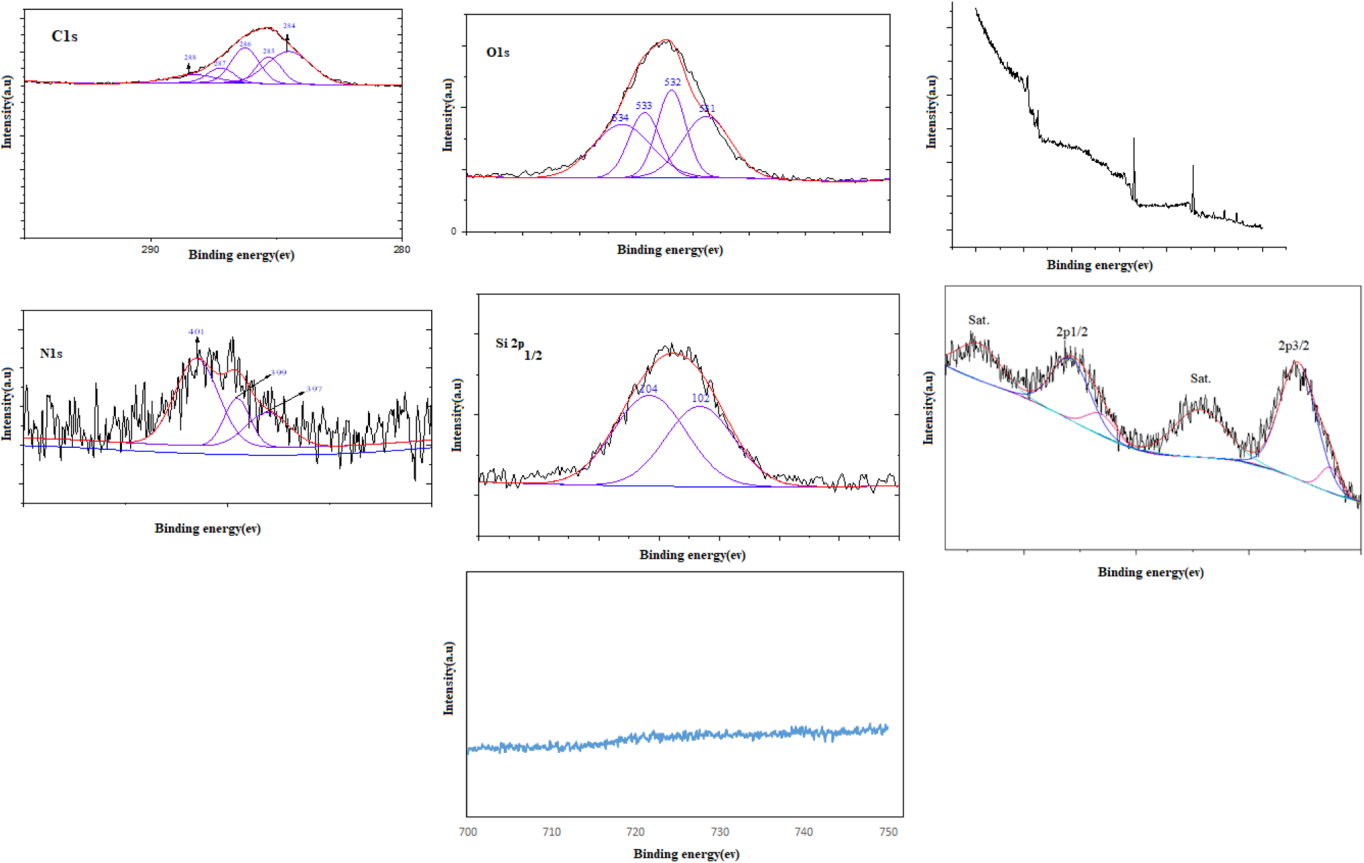
XPS spectra of the as-prepared Fe_3_O_4_@SiO_2_@SBA-3@CPTMS@Arg-Cu.

XPS analysis was performed to determine the oxidation states of Cu in the prepared nanocomposite (Fig. 4). The binding energy values of 935.6 and 955.8 eV, corresponding to the spin orbit splitting components of Cu 2p_3/2_ and Cu 2p_1/2_ in the +2 oxidation state of copper, agreed well with the reported literature values.^52^ Similarly, the peaks located at 932.8 eV and 953.1 eV corresponded to Cu 2p_3/2_ and Cu 2p_1/2_, respectively, indicating the presence of Cu compounds containing Cu^+^ ions.^53^ Besides, in Fig. 4, the Fe 2p_3/2_ spectrum displayed two minor peaks at 711.4 eV and 710.0 eV, which could be ascribed to Fe^3+^ and Fe^2+^, respectively.^54^ The deconvoluted XPS spectrum of Si displayed two sub-peaks for Si 2p (at 102 eV and 104 eV), which could be assigned to Si–C and Si–O.^55^ The deconvoluted high-resolution C 1s spectrum of the film yielded peaks at 284 eV, 285 eV, 286 eV, and 289.4 eV, which were attributed to the sp3 bulk bonded carbon C–C, C–N, C–O, and O–C=O bonds.^56–58^ The presence of the amino group in the catalyst led to the presence of an N1s peak, which was deconvoluted into three contributions, assigned to the amino group (C=N–C, 397 eV), (C=N, 399.4 eV), and (C–NH, 401 eV).^59,60^ The O 1s spectrum of the catalyst in Fig. 4 could be deconvoluted into four peaks for O–C=O at 531 eV, C–O at 533 eV, C=O at 532 eV, and H_2_O at 534 eV.^61–63^

**Fig. 5:**
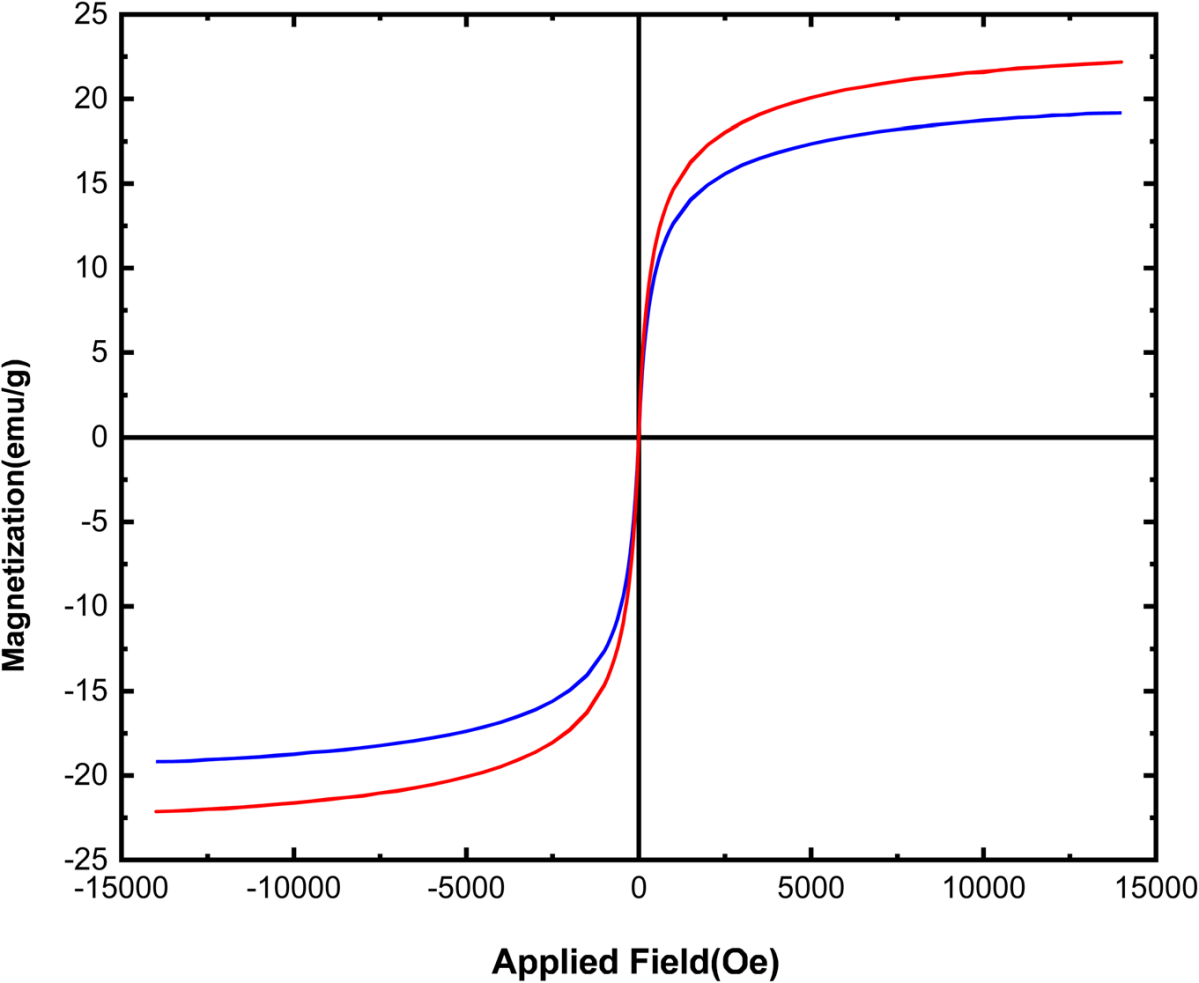
VSM patterns of Fe_3_O_4_@SiO_2_@SBA-3 (red) and Fe_3_O_4_@SiO_2_@SBA-3@CPTMS@Arg-Cu (blue).

Vibrating sample magnetometry curves for Fe_3_O_4_@SiO_2_@SBA-3 nanoparticles and Fe_3_O_4_@SBA-3@CPTMS@Arg-Cu at room temperature are shown in Fig. 5. The *M*_s_ value for Fe_3_O_4_@SiO_2_@SBA-3@CPTMS@Arg-Cu was found to be 18 emu g^−1^. The *M*_s_ value for the catalyst was lower than that of the naked one (23 emu g^−1^). This result seems to confirm that the Fe_3_O_4_@SiO_2_@SBA-3 surface was surrounded by CPTMS@Arg-Cu, which quenched the magnetic properties.^64^

Atomic absorption spectroscopy (AAS) was used to determine the exact amount of copper in Fe_3_O_4_@SiO_2_@SBA-3@CPTMS@Arg-Cu. Based on this experiment, the amount of copper in 1 g of Fe_3_O_4_@SiO_2_@SBA-3@CPTMS@Arg-Cu was found to be 2.9 × 10^−4^ mol.

**Fig. 6:**
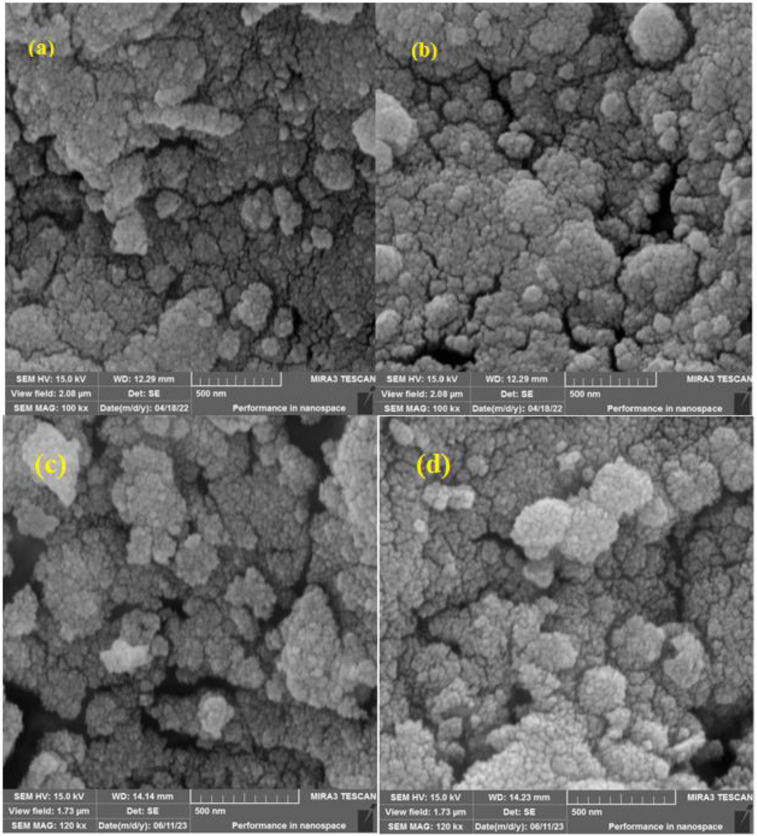
SEM images of Fe_3_O_4_@SiO_2_@SBA-3@CPTMS@Arg-Cu before recovery (a and b) and after recovery (c and d).

SEM was performed to observe the morphology of the formed Fe_3_O_4_@SiO_2_@SBA-3@CPTMS@Arg-Cu. The SEM images of Fe_3_O_4_@SiO_2_@SBA-3@CPTMS@Arg-Cu are presented in one scale: 500 nm. The retainment of the shape and regularity of the parent mesopores in the newly designated hybrid materials were confirmed in the SEM images (Fig. 6).

**Fig. 7:**
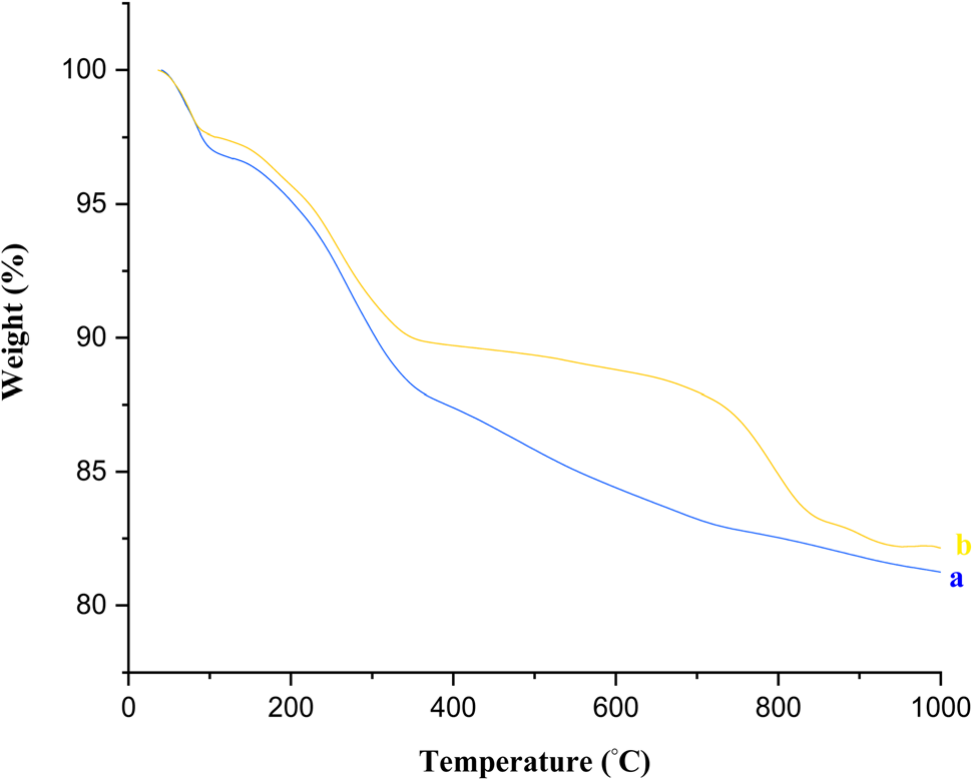
TGA curves of Fe_3_O_4_@SiO_2_@SBA-3@CPTMS@Arg-Cu before recovery (a) and after recovery (b).

Thermogravimetric analysis (TGA) can be used to study the behavior of materials *versus* temperature (Fig. 7). As shown, there was a weight loss below 250 °C corresponding to the removal of the adsorbed water and organic solvents (about 7 wt%). The second weight loss region was mainly related to the thermal decomposition of organic compounds in the temperature range between 250 °C and 700 °C (about 11 wt%). Moreover, the continuation of weight loss with increasing temperatures up to 1000 °C was ascribed to the condensation of the silanol groups of Fe_3_O_4_@SiO_2_@SBA-3.

**Fig. 8:**
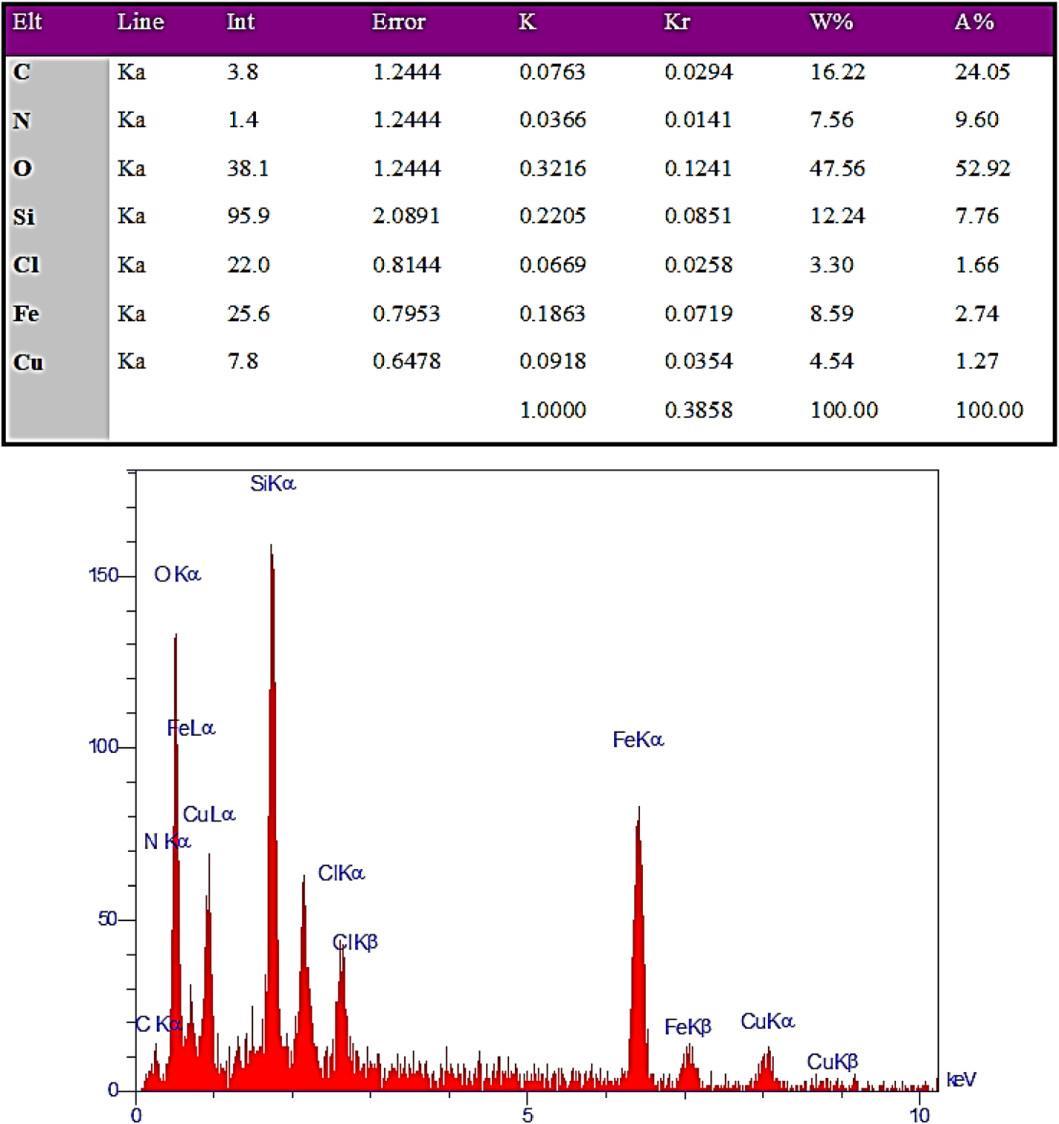
EDX spectrum of Fe_3_O_4_@SiO_2_@SBA-3@CPTMS@Arg-Cu.

Energy dispersive X-ray (EDX) was used to determine the presence and distribution of the components in the prepared nanocatalyst (Fig. 8). EDX analysis confirmed the presence of all elements (C = 16.22%, N = 7.56%, O = 47.56%, Cu = 4.54%, Si = 12.24%, and Fe = 8.59%) that had been used in the synthesis of the nanocatalyst.

**Fig. 9:**
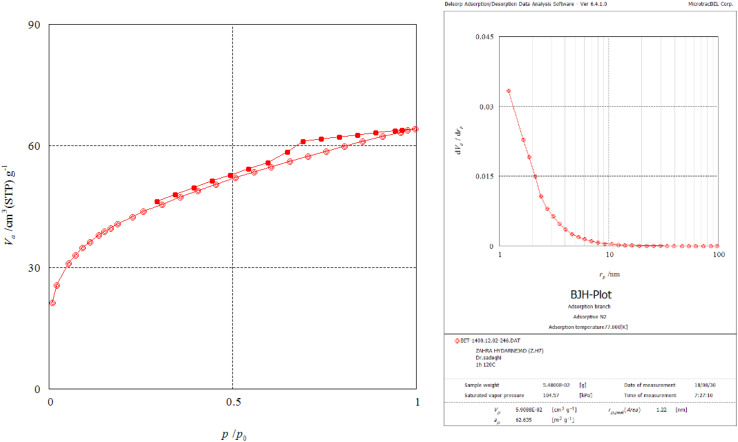
N_2_-adsorption isotherms of Fe_3_O_4_@SiO_2_@SBA-3@CPTMS@Arg-Cu.

The N_2_ adsorption–desorption isotherm and BJH pore size distribution curve of the Fe_3_O_4_@SiO_2_@SBA-3@CPTMS@Arg-Cu are shown in Fig. 9. The nitrogen adsorption–desorption isotherm of the catalyst after modification exhibited isotherms of type IV(a) characteristic of porous materials, with the hysteresis loop classified according to the IUPAC convention as H2(b).^65^ The BET surface area was found to be 145 m^2^ g^−1^. The average pore diameter of the catalyst pores was 2.68 nm. The total pore volume of the catalyst was approximately 0.1 cm^3^ g^−1^, which was estimated at a relative pressure of 0.99 (Table 1). The average pore size obtained by the BJH method using the adsorption branch was 1.22 nm (Table 2).

**Table 1:** Nitrogen adsorption–desorption data for Fe_3_O_4_@SiO_2_@SBA-3@CPTMS@Arg-Cu (from BET calculation)

Entry	BET plot
*V*_m_	34 cm^3^ g^−1^
*a*_s,BET_	145 m^2^ g^−1^
Total pore volume (*p*/*p*_0_ = 0.990)	0.099 cm^3^ g^−1^
Mean pore diameter	2.68 nm

**Table 2:** BJH-plot data for Fe_3_O_4_@SiO_2_@SBA-3@CPTMS@Arg-Cu

Plot data	Adsorption branch
*V*_p_	0.05 cm^3^ g^−1^
*r*_p_, peak (area)	1.22 nm
*a*_p_	62.635 m^2^ g^−1^

### 3.1 Catalytic studies

After the preparation and characterization of the designed magnetic mesoporous material, the catalytic activity of this compound was investigated in the hydrolysis of nitriles to amides. Initially, benzonitrile was chosen as the model substrate to optimize the reaction parameters such as the amount of catalysts and the types of base and solvent, and the temperature (Table 3). The effect of the solvent was examined, and it was observed that the reaction was highly effective in water. The control experiment confirmed that the reaction did not occur in the presence of CuCl, Fe_3_O_4_, Fe_3_O_4_@SiO_2_, Fe_3_O_4_@SiO_2_@SBA-3, Fe_3_O_4_@SiO_2_@SBA-3@Arg, or Arg. Furthermore, the progress of the reaction depended on the amount of catalyst; whereby the reaction could be completed in the presence of 40 mg of this catalyst. The ideal temperature for the reaction was found to be 75 °C.

**Table 3:** Optimization of the reaction conditions for the hydrolysis of nitriles to amides in the presence of Fe_3_O_4_@SiO_2_@SBA-3@CPTMS@Arg-Cu

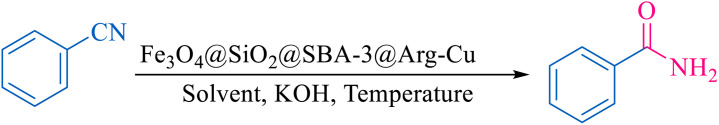						
Entry^a^	Solvent	Catalyst (mg)	Amount of KOH (mmol)	Temperature (°C)	Time (min)	Yield^b^ (%)
1	Propanol	60	3	75	180	68
2	H_2_O	60	3	75	180	97
3	EtOH	60	3	75	180	75
4	MeOH	60	3	Reflux	180	40
5	THF	60	3	Reflux	180	N. R
6	H_2_O	60	4	75	180	98
7	H_2_O	60	2	75	150	97
8	H_2_O	50	2	75	150	98
9	H_2_O	40	2	75	150	97
10	H_2_O	40	2	95	150	94
11	H_2_O	40	2	55	150	83
12^c^	H_2_O	60	3	75	180	57
13^d^	H_2_O	60	3	75	180	N.R
14^e^	H_2_O	60	3	75	180	N.R
15^f^	H_2_O	60	3	75	180	N.R
16^g^	H_2_O	60	3	75	180	N.R
17^h^	H_2_O	60	3	75	180	N.R

a Reaction conditions: benzonitrile (1 mmol), base (mmol), solvent (2 mL), and temperature.

b Isolated yield.

c CuCl.

d Fe_3_O_4_.

e Fe_3_O_4_@SiO_2_.

f Fe_3_O_4_@SiO_2_@SBA-3.

g Fe_3_O_4_@SiO_2_@SBA-3@Arg.

h Arg alone.

After optimization of the reaction conditions, the limitations and generality of this work were investigated. Therefore, various benzonitrile-containing electron-withdrawing substituents were examined. The experimental results showed that the desired product was obtained with good yields (Table 4).

**Table 4:** Catalytic hydrolysis of nitriles to amides in the presence of Fe_3_O_4_@SiO_2_@SBA-3@CPTMS@Arg-Cu

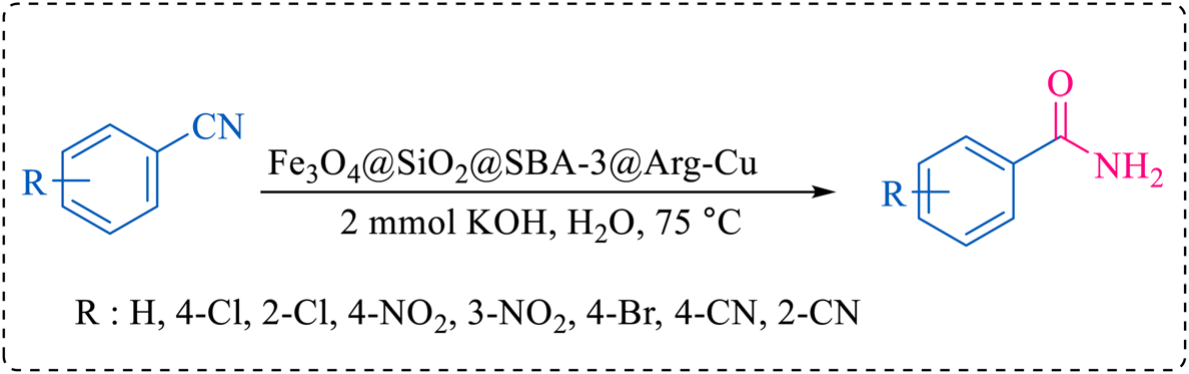						
Entry^a^	Nitrile	Product	Time (min)	Yield^b^ (%)	M.p. (°C)	
					Found	Reported
1	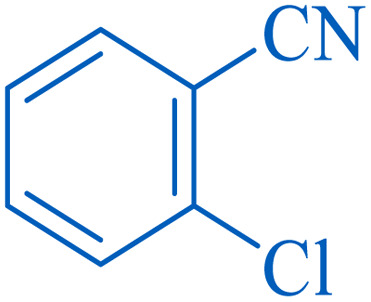	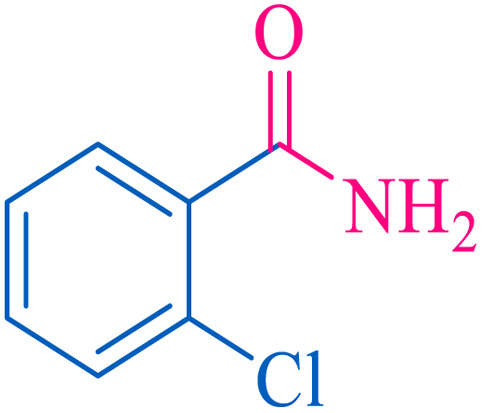	30	94	134–136	138–140 (ref. 66)
2	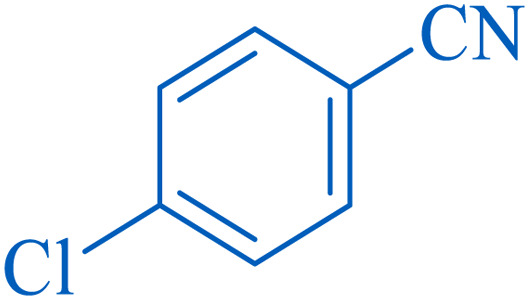	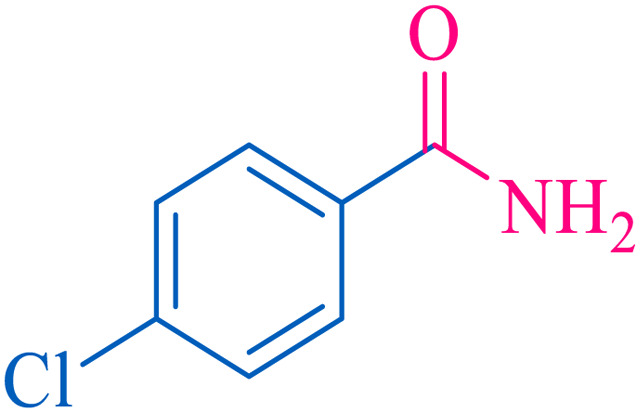	90	92	171–172	171–172 (ref. 66)
3	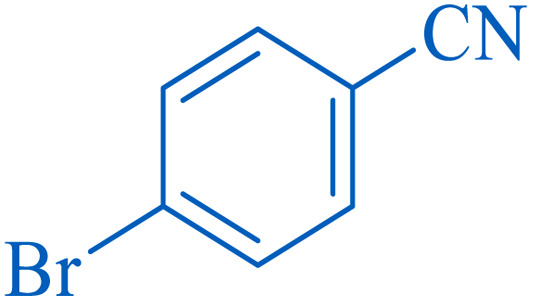	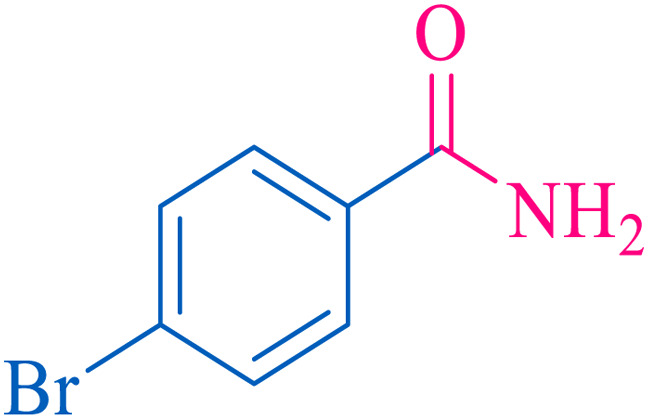	105	90	189–190	191–192 (ref. 66)
4	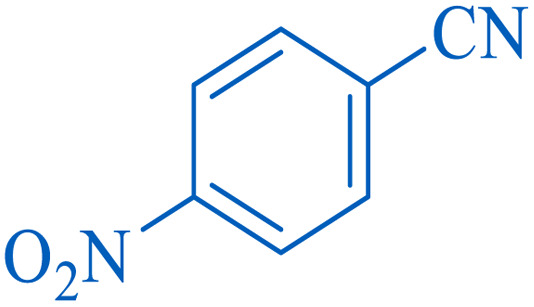	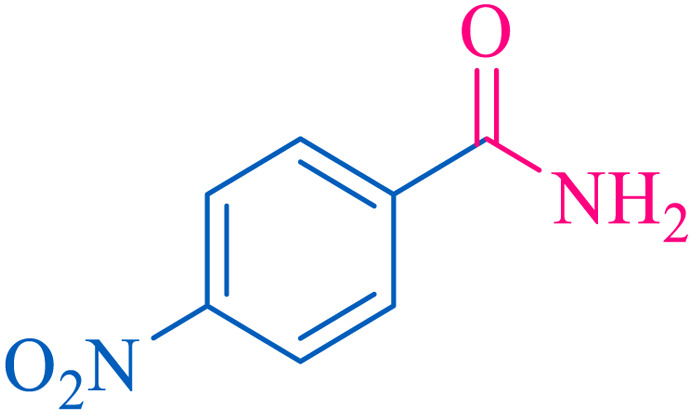	95	91	200–202	199–200 (ref. 66)
5	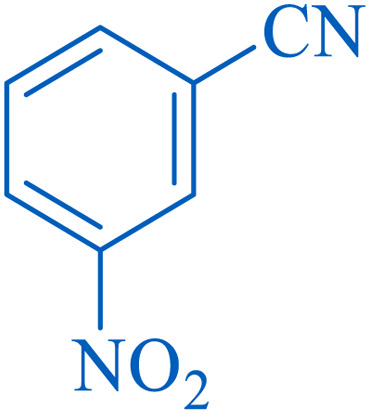	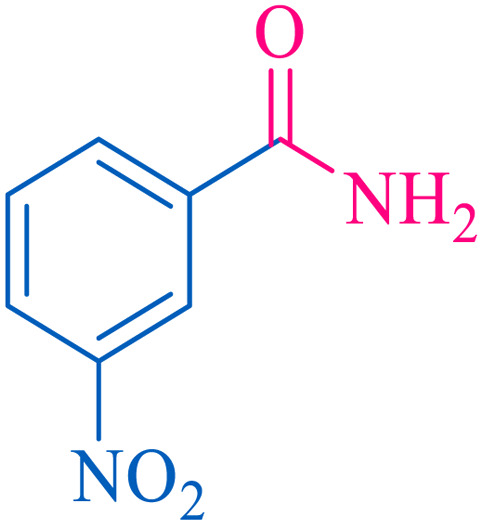	50	89	143–144	144–145 (ref. 67)
6	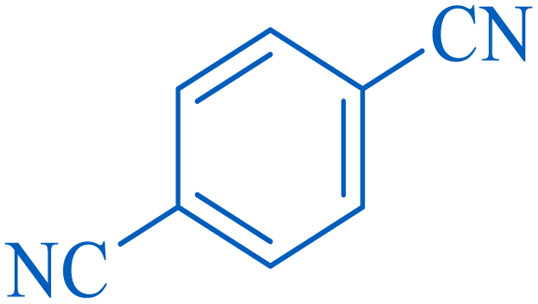	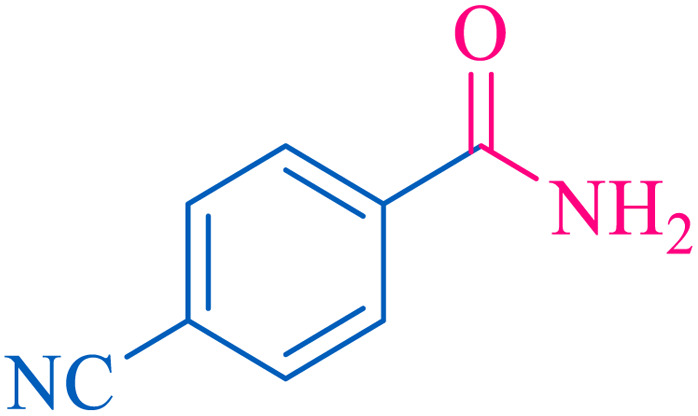	60	83	223–225	221–223 (ref. 66)
7	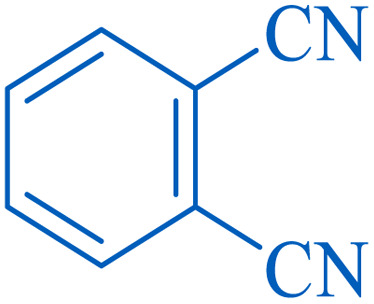	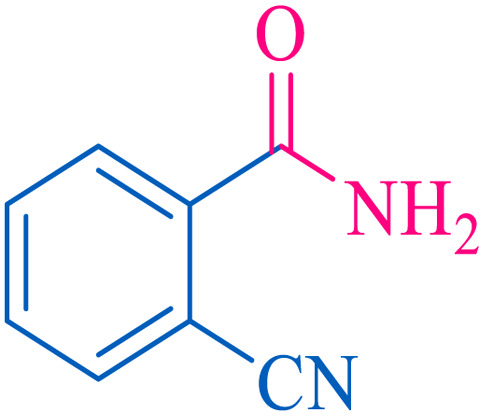	75	85	224–226	223–227 (ref. 68)
8	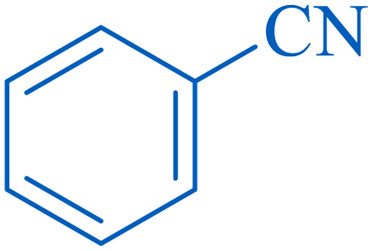	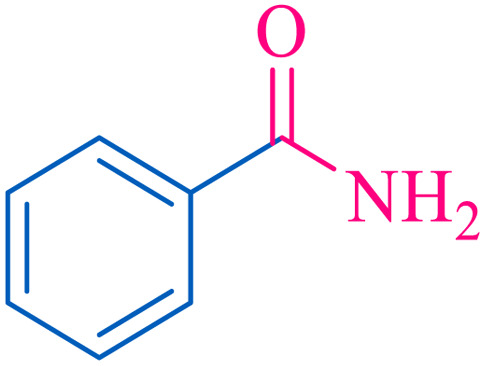	150	97	126–127	126–127 (ref. 66)

a Reaction conditions: nitrile (1 mmol), base (2 mmol), catalyst (40 mg), H_2_O (2 mL), at 75 °C.

b Isolated yield.

**Scheme 2:**
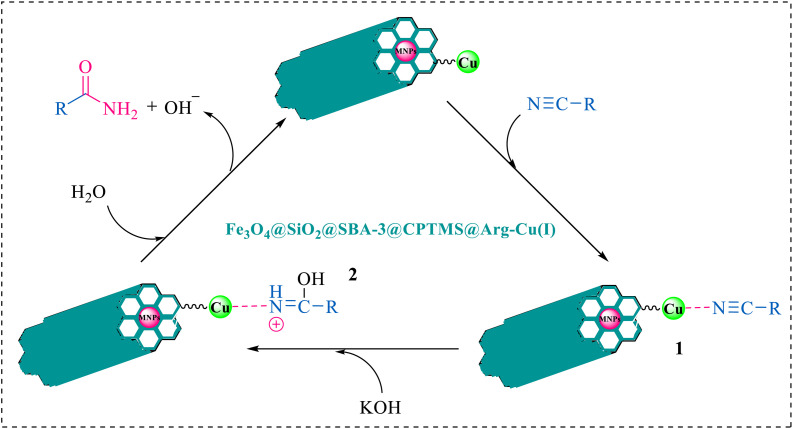
Proposed mechanism for the hydrolysis of nitriles to amides.

The proposed mechanism of the reaction is shown in Scheme 2. The first step involves the coordination of the nitrile nitrogen atoms with copper to form complex 1 and its nucleophilic attack by KOH.

In the second part, the catalytic activity of this catalyst and the synthesis of 5-substituted 1*H*-tetrazole were studied as well. Initially, the reaction of benzonitrile with sodium azide was selected as a model reaction, and different parameters including the solvent type and temperature were studied. The results are summarized in Table 5. First, the effects of different solvents and various temperatures on the model reaction were investigated. According to the data given in (Table 5), PEG was the most efficient solvent for this reaction. When the reaction was conducted at 80 °C and 100 °C, the observed yields were very low (Table 5). The ideal temperature for the reaction was found to be 120 °C.

**Table 5:** Optimization of the reaction conditions for the synthesis of 5-substituted 1*H*-tetrazoles in the presence of Fe_3_O_4_@SiO_2_@SBA-3@CPTMS@Arg-Cu

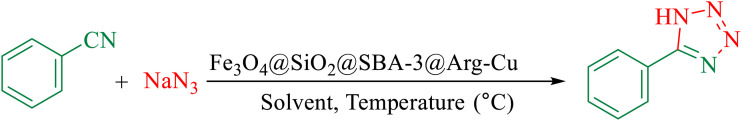					
Entry^a^	Solvent	Catalyst (mg)	Temperature (°C)	Time (min)	Yield^b^ (%)
1	PEG	50	120	100	95
2	DMF	50	120	100	59
3	DMSO	50	120	100	68
4	H_2_O	50	Reflux	100	Trace
5	PEG	30	120	100	78
6	PEG	40	120	100	84
7	PEG	50	100	100	83
8	PEG	50	80	100	68

a Reaction conditions: benzonitrile (1 mmol), sodium azide (1.2 mmol), solvent (2 mL), and temperature.

b Isolated yield.

To understand the scope and effectiveness of the catalyst, different substituted benzonitriles (Table 6) were selected to participate in the described cycloaddition reaction. The results showed that a wide range of nitrile compounds could produce the corresponding tetrazoles in excellent isolated yields.

**Table 6:** Catalytic synthesis of 5-substituted 1*H*-tetrazoles in the presence of Fe_3_O_4_@SiO_2_@SBA-3@CPTMS@Arg-Cu

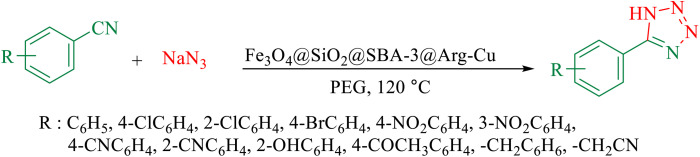						
Entry^a^	Nitrile	Product	Time (min)	Yield^b^ (%)	M.p. (°C)	
					Found	Reported
1	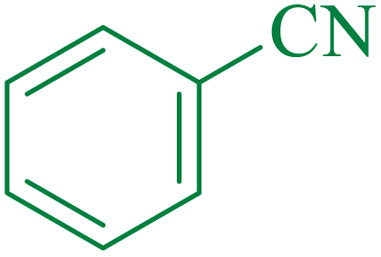	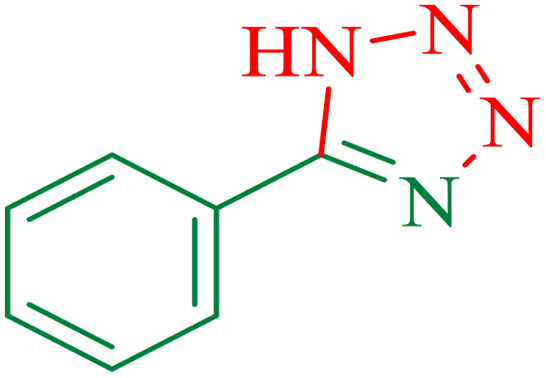	100	95	212–216	212–213 (ref. 69)
2	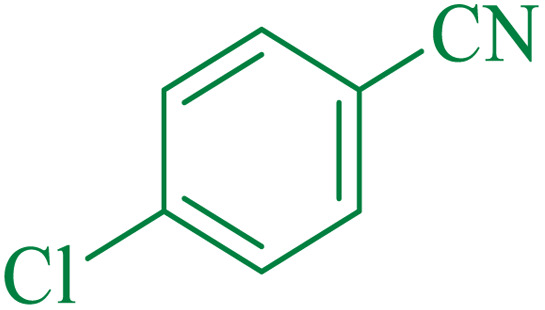	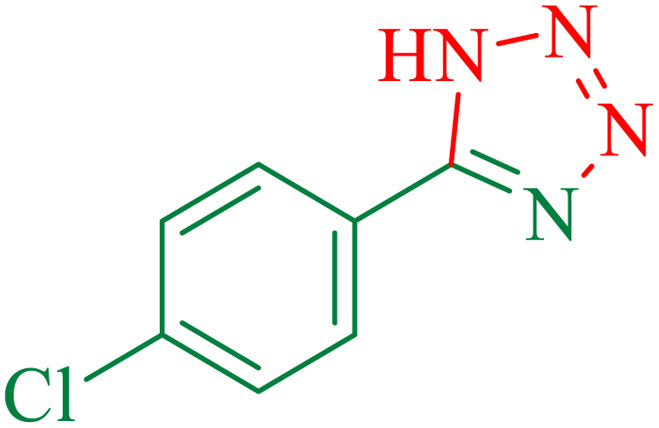	83	96	260–262	261–264 (ref. 69)
3	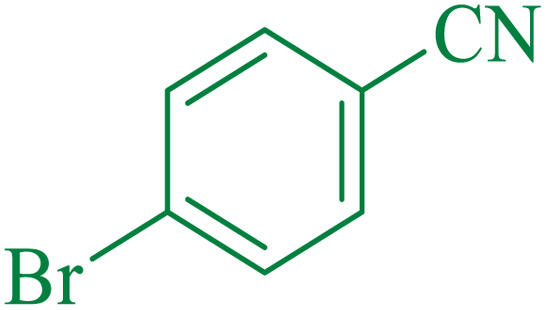	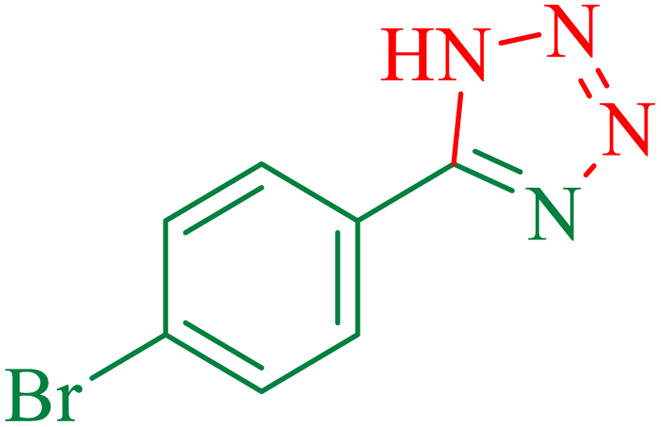	95	95	266–267	264–265 (ref. 70)
4	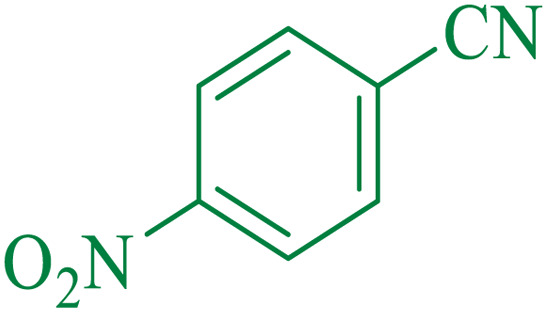	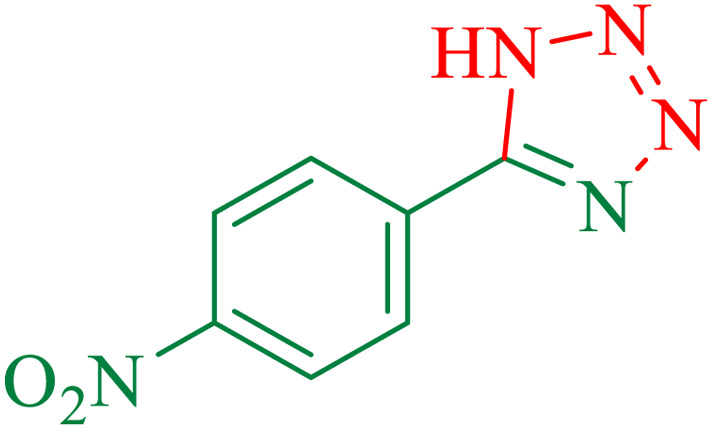	35	93	216–218	217–219 (ref. 70)
5	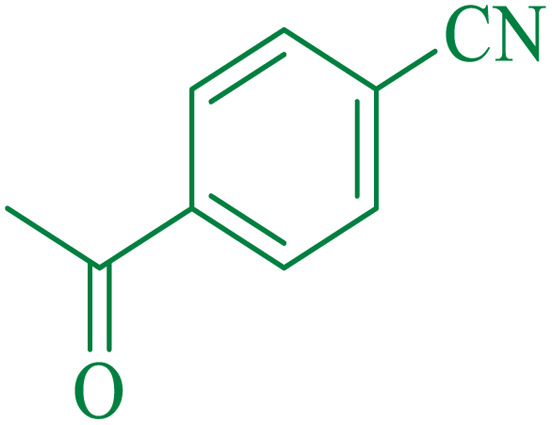	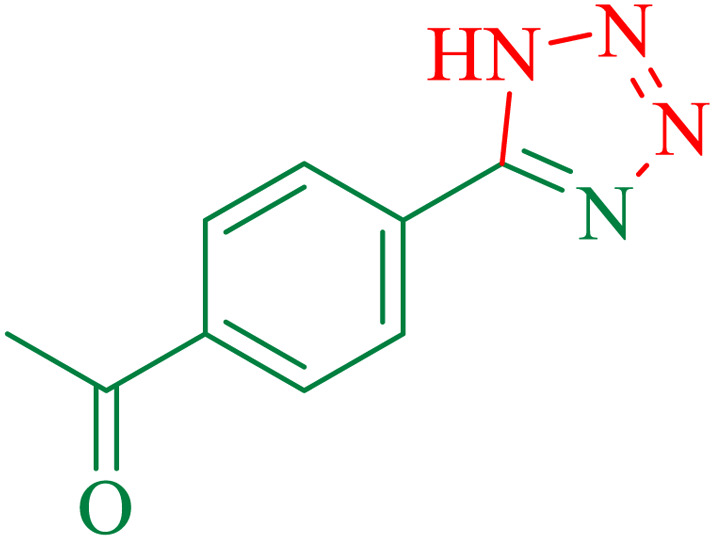	170	91	174–175	175–177 (ref. 33)
6	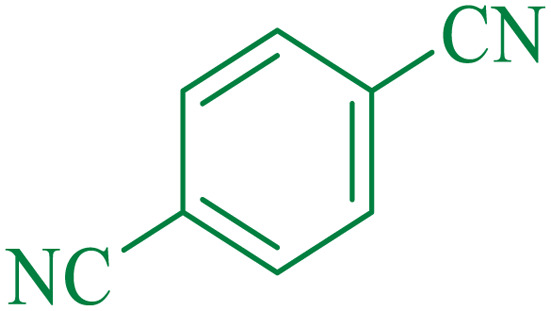	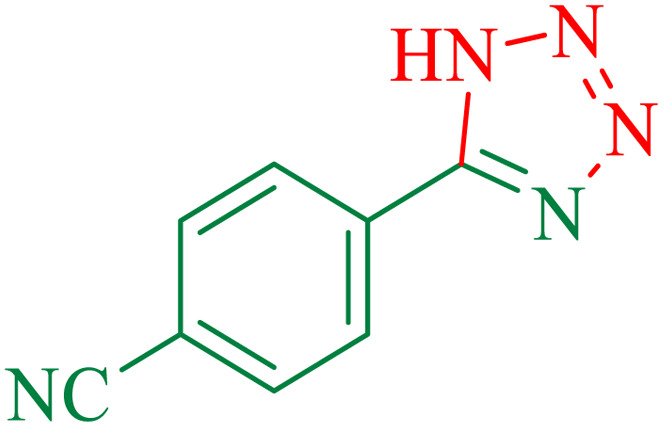	32	90	255–257	251–253 (ref. 69)
7	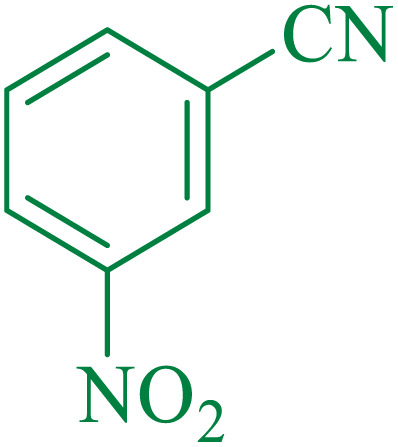	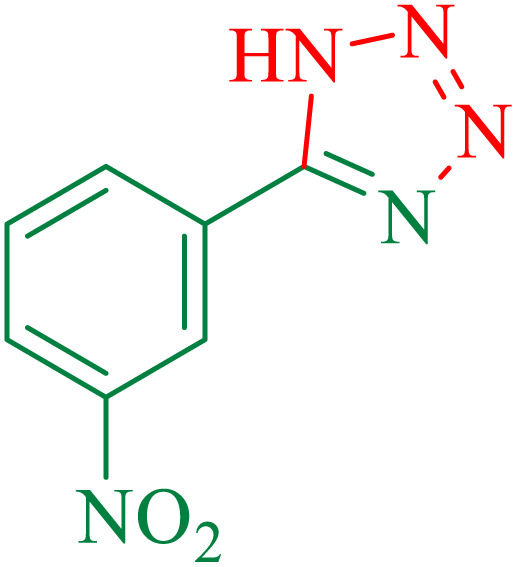	87	91	150–151	148–151 (ref. 69)
8	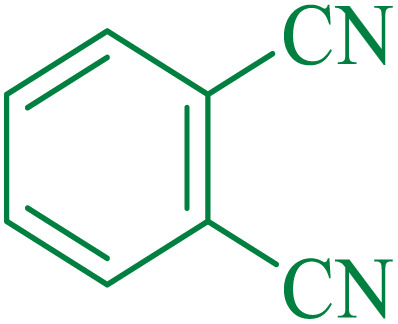	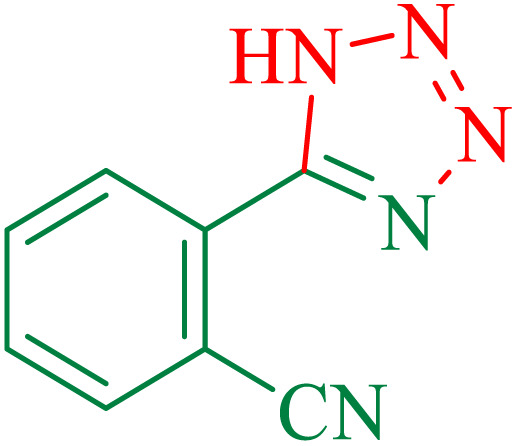	20	92	209–211	209–213 (ref. 71)
9	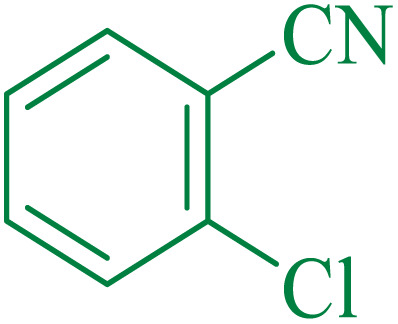	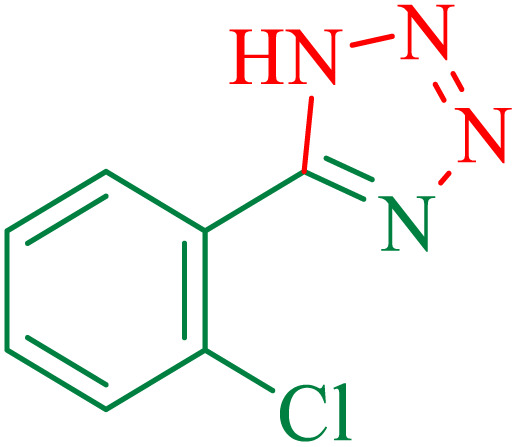	110	90	181–183	182–183 (ref. 69)
10	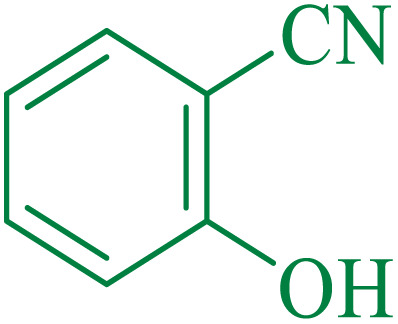	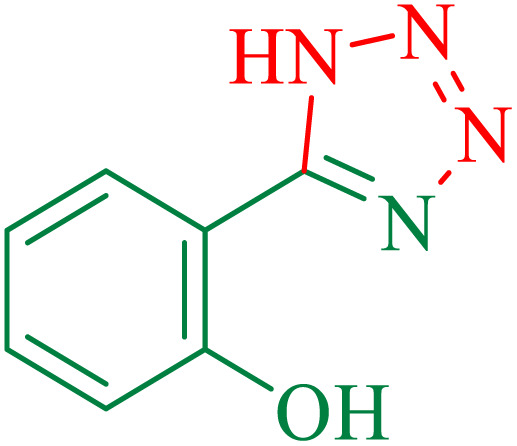	28	89	220–223	220–222 (ref. 71)
11	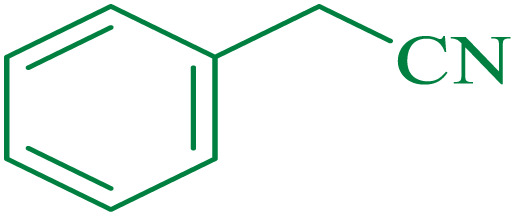	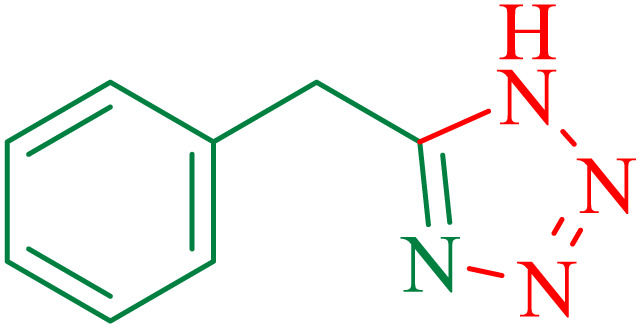	55	85	117–119	117–120 (ref. 70)
12	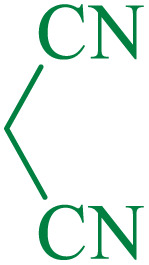	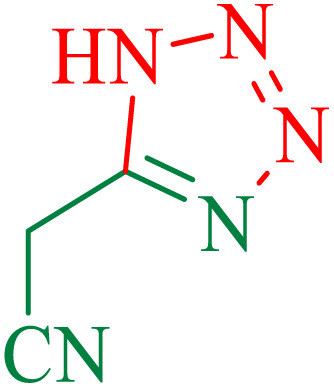	38	88	115–117	113–115 (ref. 72)

a Reaction conditions: nitrile (1 mmol), sodium azide (1.2 mmol), catalyst (50 mg), and PEG (2 mL), at 120 °C.

b Isolated yield.

**Scheme 3:**
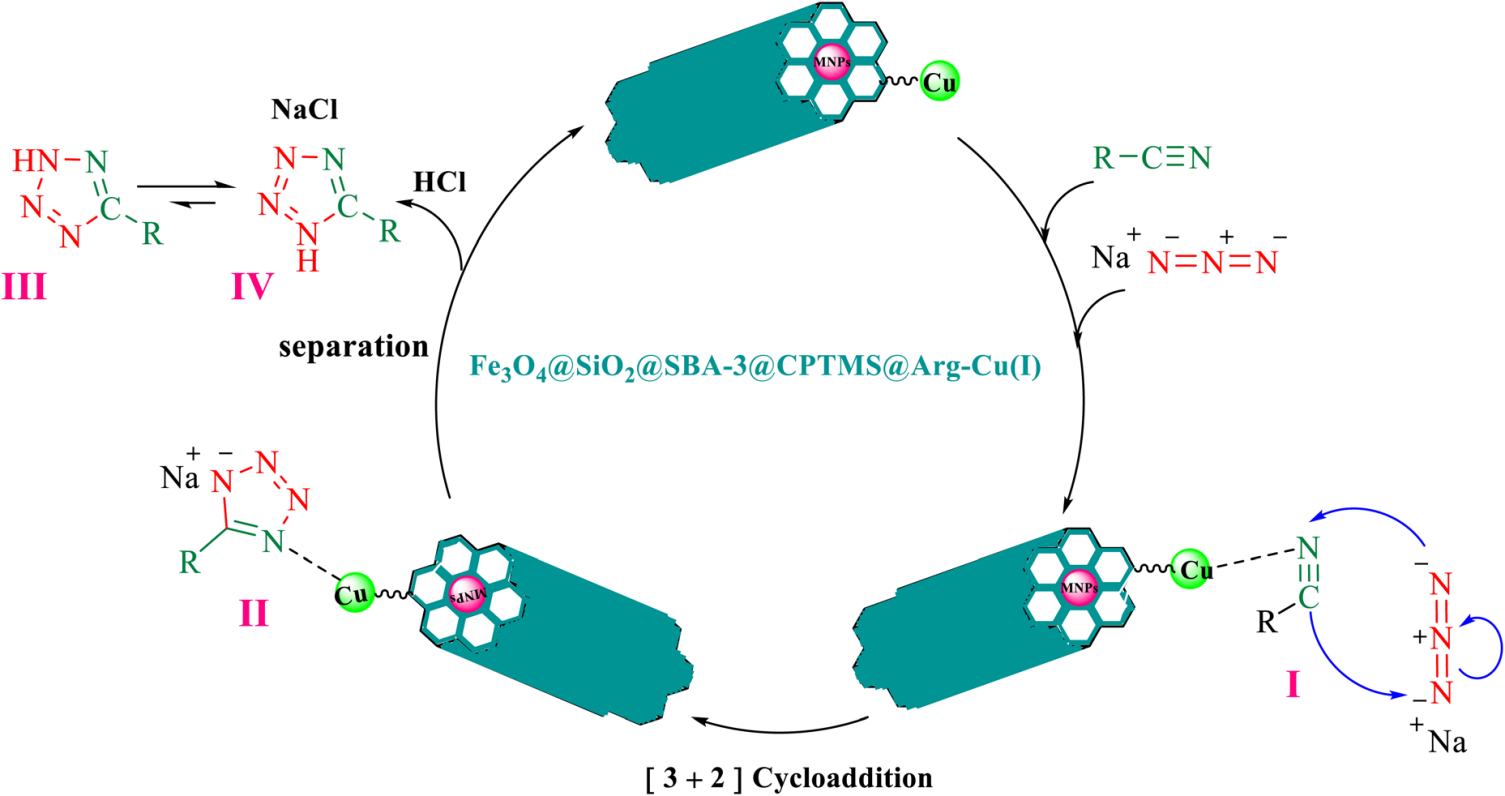
Proposed mechanism for the synthesis of 5-substituted 1*H*-tetrazoles.

The proposed mechanism of the reaction is shown in Scheme 3. The first step involves the coordination of the nitrile nitrogen atoms with copper to form complex I, which accelerates the cyclization step. Next, the [3 + 2] cycloaddition occurs between the two metal-coordinated CN bonds of the nitrile compound and azide ion to form intermediate II. Finally, the acidic work-up affords the desired product III.

### 3.2 Catalytic stability and reusability

**Fig. 10:**
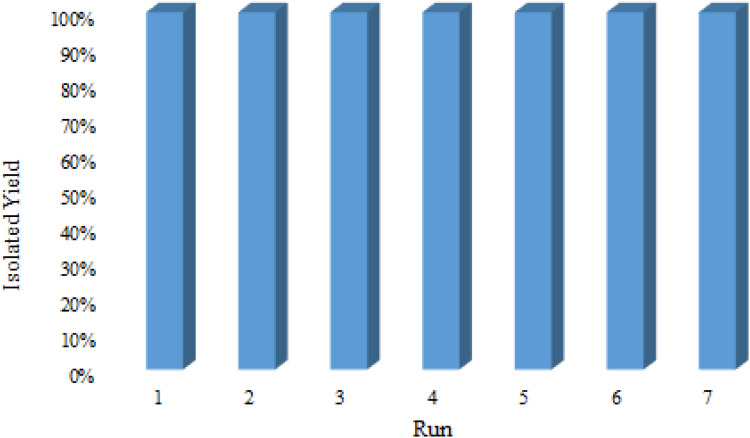
Recyclability of Fe_3_O_4_@SiO_2_@SBA-3@CPTMS@Arg-Cu NPs for the hydrolysis of benzonitrile to benzamide.

The rapid separation and efficient recycling of catalysts after a catalytic reaction are considered to be important requirements, along with high catalytic performances. For this purpose, the recovery and recyclability of the employed Fe_3_O_4_@SiO_2_@SBA-3@CPTMS@Arg-Cu NPs were considered for the hydrolysis of benzonitrile to benzamide. Upon completion of the reaction, the catalyst was recovered *via* an external magnet, and then washed thoroughly with ethyl acetate, and dried under vacuum conditions. The metal leaching of the catalyst was studied by a hot filtration test. To examine the leaching of the copper in the reaction mixture and the heterogeneity of the described catalyst, we performed a hot filtration experiment for the synthesis of 5-substituted 1*H*-tetrazoles. In this study, we found that the yield of the product at half the reaction time was 55%. Then, the reaction was repeated, and at half the reaction time, the catalyst was separated and the filtrate was allowed to react further. The yield of the reaction after this test was 55%, which confirmed that leaching of copper had not occurred. The recovered catalyst was then applied in the next cycle. The results indicated that this catalyst could be successfully reused seven times without any significant loss of activity (Fig. 10). To demonstrate the structural stability of the catalyst after recycling, the recovered catalyst was characterized using FT-IR, TGA, SEM, and XRD techniques. The recovered catalyst was investigated using XRD (Fig. 9), and FT-IR (Fig. 10). To confirm the stability of the catalytic system, the recovered catalyst was characterized using FT-IR, TGA, SEM, and XRD techniques (Fig. 1, 3, 6, and 7, respectively). TGA data showed the weight change in the recovered catalyst compared to the fresh catalyst. The characterization also showed that the catalytic system could be reused for several runs without any change in its structure. Moreover, the XRD pattern for the Cu-based catalyst shows a decrease in intensity due to the destroyed crystal structure and the size distribution of copper nanoparticles in the reactivated catalyst.^73^

### 3.3 Comparison of the catalyst behavior with previously reported procedures

We evaluated the efficiency of the described catalytic system for comparison with previously reported procedures in the literature. A comparison of the results showed a better catalytic activity of the Fe_3_O_4_@SiO_2_@SBA-3@CPTMS@Arg-Cu NPs for the hydrolysis of benzonitrile to benzamide (Table 7). The comparison indicated that the prepared magnetite mesoporous material as a versatile nanocatalyst was comparable to or maybe better in some aspects than the other reported catalysts.

**Table 7:** Comparison of the performance of the Fe_3_O_4_@SiO_2_@SBA-3@CPTMS@Arg-Cu NP catalyst and other catalysts for the hydrolysis of benzonitrile to benzamide

Entry	Catalyst	Conditions	Time (h)	Yield^a^ (%)	Ref.
1	TBANa-SrPd_12_(pent)_3_	DMF, H_2_O, 90 °C	600	88	74
2	Fe_3_O_4_@SiO_2_-NHC-Cu	H_2_O, 110 °C	360	92	68
3	Ru@imine-nanoSiO_2_	KOH, i-PrOH, 40 °C, air	300	75	75
4	Au NPs	KOH, i-PrOH : H_2_O (1 : 1), 80 °C	180	99	76
5	Complex [OsCl_2_(η^6^-*p*-cymene){PPh_2_(NMe_2_)}]	H_2_O, 100 °C	180	97	77
6	Fe_3_O_4_@SiO_2_@SBA-3@CPTMS@Arg-Cu	KOH, H_2_O, 75 °C	150	97	This work

a Isolated yield.

## 4. Conclusions

In conclusion, we developed a simple, efficient, and environmentally benign method for the hydrolysis of nitriles to amides and the synthesis of 5-substituted 1*H*-tetrazoles, employing Fe_3_O_4_@SiO_2_@SBA-3@CPTMS@Arg-Cu as a recyclable heterogeneous catalyst with a magnetic and mesoporous nature. The advantages of the present protocol are the reusability of the catalyst for up to four cycles, excellent product yield, shorter reaction time, and green reaction conditions.

## Conflicts of interest

The authors declare that they have no known competing financial interests or personal relationships that could have influenced the work reported in this study.

## Supplementary Material

NA-006-D3NA00318C-s001
