# Supplementary material for: Fe3O4@SiO2@SBA-3@CPTMS@Arg-Cu: preparation, characterization, and catalytic performance in the conversion of nitriles to amides and the synthesis of 5-substituted 1H-tetrazoles
Source: Nanoscale Adv. 2024 Mar 26;6(9):2431–46. doi: 10.1039/d3na00318c (PMC11059512; doi:10.1039/d3na00318c)
Supplement: NA-006-D3NA00318C-s001 [file NA-006-D3NA00318C-s001.pdf]

Electronic Supplementary Material (ESI) for Nanoscale Advances.
This journal is © The Royal Society of Chemistry 2023

Fe_3O_4@SiO_2@SBA-3@CPTMS@Arg-Cu: preparation, characterization, and catalytic performance for the conversion of nitriles to amides and synthesis of 5-substituted 1H-tetrazoles

Zahra Heidarnezhad, [a] Arash Ghorbani-Choghamarani,[b,]* Zahra Taherinia [a]

Selected Spectral Data

2-Chlorobenzamide (Table 4, entry 1): ^{1}H NMR (400 MHz, DMSO) δ 7.98(s, 2H), 7.48-7.39(m, 4H)). ^{13}C NMR (100 MHz, DMSO-d_6, ppm) δ 168. 6, 162.2, 137.4, 130.9, 130.0, 129.0, 127.4.

4-Chlorobenzamide (Table 4, entry 2): ^{1}H NMR (400 MHz, DMSO) δ 8.0 (s, 2H), 7.89 (d, *J* = 8 Hz, 2H), 7.87 (d, *J* = 8 Hz, 2H). ^{13}C NMR (100 MHz, DMSO-d_6, ppm) δ 167.2, 162.5, 136.4, 133.4, 129.7, 128.6.

4-Bromobenzamide (Table 4, entry 3): ^{1}H NMR (400 MHz, DMSO) δ 8.0 (s, 2H), 7.82 (d, *J* = 6 Hz, 2H), 7.65 (d, *J* = 8 Hz, 2H). ^{13}C NMR (100 MHz, DMSO-d_6, ppm) δ 166.9, 133.4, 131.2, 129.6, 124.9.

4-Nitrobenzamide (Table 4, entry 4): ^{1}H NMR (400 MHz, DMSO) δ 8.3 (d, *J* = 12 Hz, 2H), 8.0 (d, *J* = 8 Hz, 2H), 7.7 (s, 1H).

Benzamide (Table 4, entry 8): ^{1}H NMR (400 MHz, DMSO) δ 7.98(s, 2H), 7.82(d, *J* = 6 Hz, 2H), 7.50-7.45(m, 3H).^{13}C NMR (100 MHz, DMSO-d_6, ppm) δ 168.3, 162.2, 134.6, 131.6, 128.6, 127.8.

5-phenyl-1H-tetrazole (Table 6, entry 1): ^{1}H NMR (400 MHz, DMSO) δ 8.03(d, *J* = 6 Hz, 3H), 7.57(d, *J* = 6 Hz, 2H).^{13}C NMR (100 MHz, DMSO-d_6, ppm) δ 162.2, 155.1, 131.6, 129.7, 127.3, 124.5.

5-(4-Chlorophenyl)-1H-tetrazole (Table 6, entry 2): ^{1}H NMR (400 MHz, DMSO) δ 8.05 (d, *J* = 8.4 Hz, 2H), 7.69 (d, *J* = 8.4 Hz, 2H). ^{13}C NMR (100 MHz, DMSO-d_6, ppm) δ 162.1, 155.3, 148.5, 133.2, 131.4, 126.4, 125.8, 121.7.

5-(4-Bromophenyl)-1H-tetrazole (Table 6, entry 3): ^{1}H NMR (400 MHz, DMSO) δ 7.95(d, *J* = 6, 2H), 7.78(d, *J* = 6, 2H). ^{13}C NMR (100 MHz, DMSO-d_6, ppm) δ 162.2, 155.3, 132.8, 129.2, 125.0, 123.9.

5-(4-nitrophenyl)-1H-tetrazole (Table 6, entry 4): ^{1}H NMR (400 MHz, DMSO) δ 8.35(d, *J* = 6 Hz, 2H), 8.22(d, *J* = 6 Hz, 2H). ^{13}C NMR (100 MHz, DMSO-d_6, ppm) δ 162.2, 155.8, 148.9, 130.9, 128.4, 124.8.

1-(4-(1H-tetrazol-5-yl)phenyl)ethan-1-one(Table 6, entry 5): ^{13}C NMR (100 MHz, DMSO-d$_6$, ppm) δ 197.3, 138.5, 129.1, 128.4, 127.1, 26.8.

4-(1H-tetrazol-5-yl)benzonitrile (Table 6, entry 6): ^{1}H NMR (400 MHz, DMSO) δ 8.20(d, *J* = 12 Hz, 2H), 8.08(d, *J* = 12 Hz, 2H). ^{13}C NMR (100 MHz, DMSO-d$_6$, ppm) δ 166.7, 162.2, 155.9, 133.7, 129.1, 127.9, 118.5, 113.7.

5-(3-nitrophenyl)-1H-tetrazole (Table 6, entry 7): δ 8.74(s, 1H), 8.41(q, *J* = 8, 2H), 7.86(t, *J* = 8, 1H).^{13}C NMR (100 MHz, DMSO-d$_6$, ppm) δ 162.1, 155.3, 148.5, 133.2, 131.4, 126.4, 125.8, 121.7.

2-(1H-tetrazol-5-yl)benzonitrile (Table 6, entry 8): ^{1}H NMR (400 MHz, DMSO) δ 8.06(s, 2H),

7.92(t, *J*=6, 1H), 7.77(t, J=6, 1H). ^{13}C NMR (100 MHz, DMSO-d$_6$, ppm) δ 162.2, 135.3, 134.1, 131.7, 130.0, 127.8, 117.6, 110.5.

2-(1H-Tetrazol-5-yl) phenol (Table 6, entry 10): ^{1}H NMR (400 MHz, DMSO) δ 8.00-7.98 (m, 1H), 7.44 (d, *J*=8, 1H), 7.40 (t, *J*=4, 1H), 7.08-6.97 (m, 1H). ^{13}C NMR (100 MHz, DMSO-d$_6$, ppm) δ 162.5, 155.7, 152.0, 133.0, 129.4, 120.0, 116.7, 110.8.

5-benzyl-1H-tetrazole (Table 6, entry 11): ^{1}H NMR (400 MHz, DMSO) δ 8.04(s, 1H), 7.31(m, 5H), 4.28(s, 2H). ^{13}C NMR (100 MHz, DMSO-d$_6$, ppm) δ 155.7, 136.2, 131.6, 129.7, 129.1, 129.0, 127.3, 29.2.

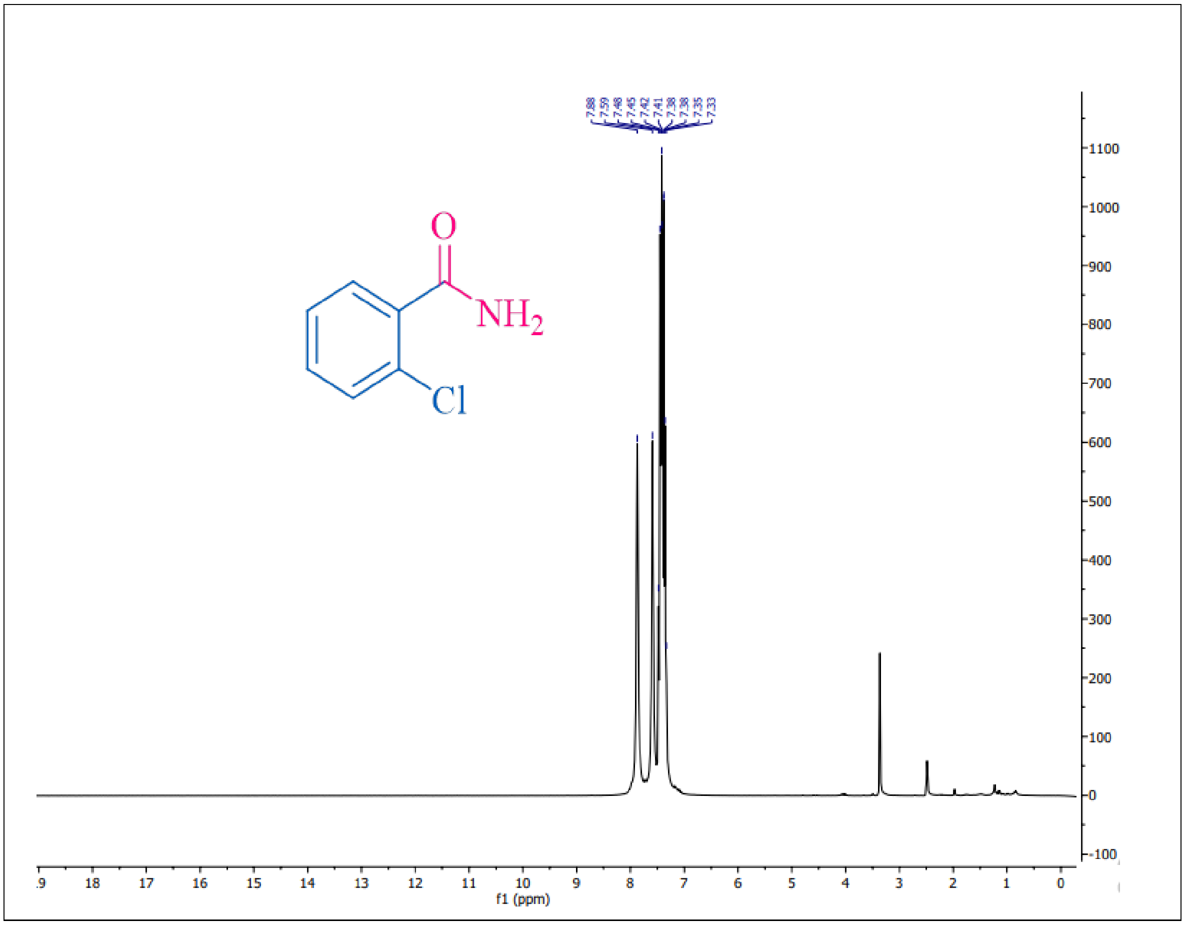
7.88
7.59
7.48
7.45
7.42
7.41
7.38
7.38
7.35
7.33
O
NH2
Cl
f1 (ppm)

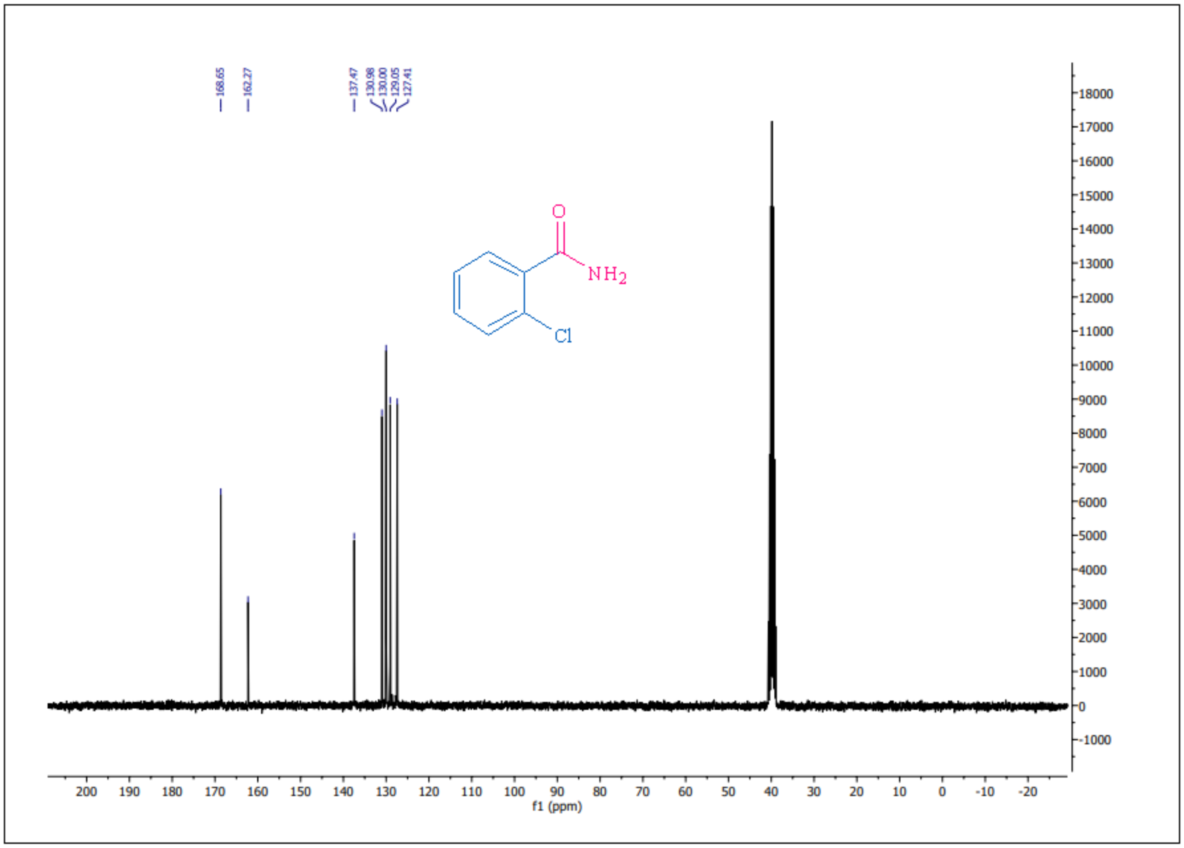

168.65
162.27
137.47
130.98
130.00
129.05
127.41
O
NH2
Cl
f1 (ppm)

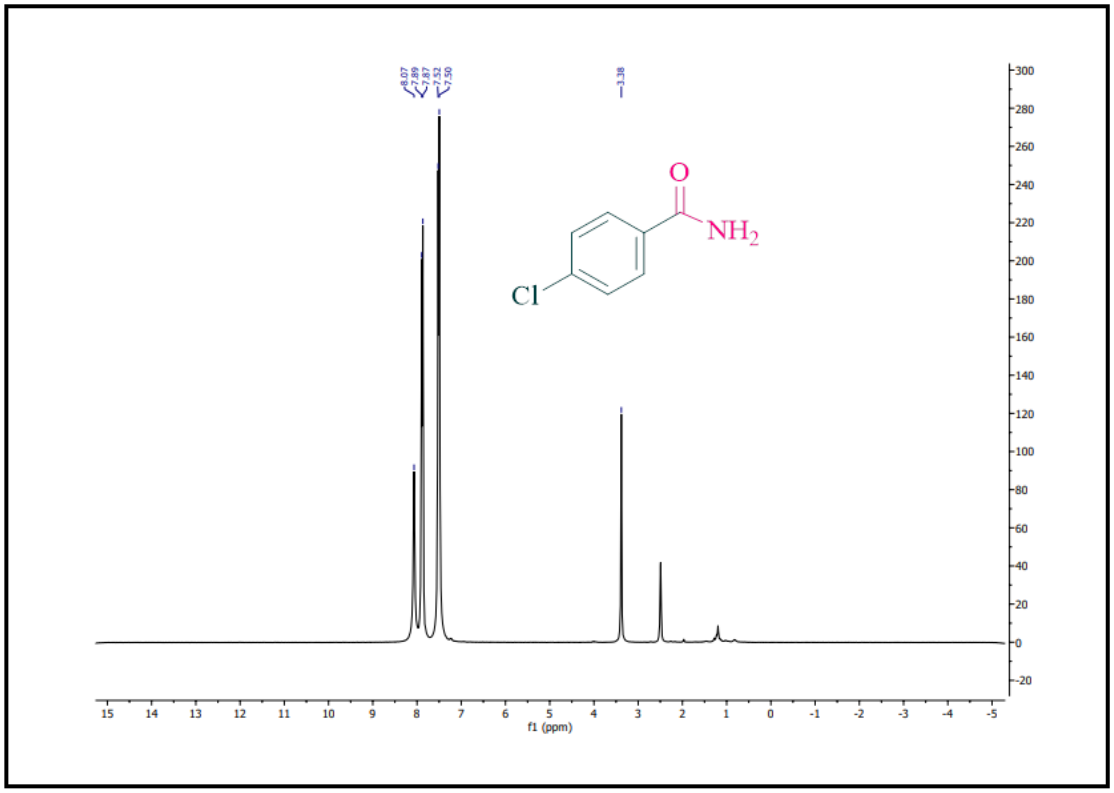
8.07
7.89
7.87
7.52
7.50
3.38
O
NH2
Cl
f1 (ppm)

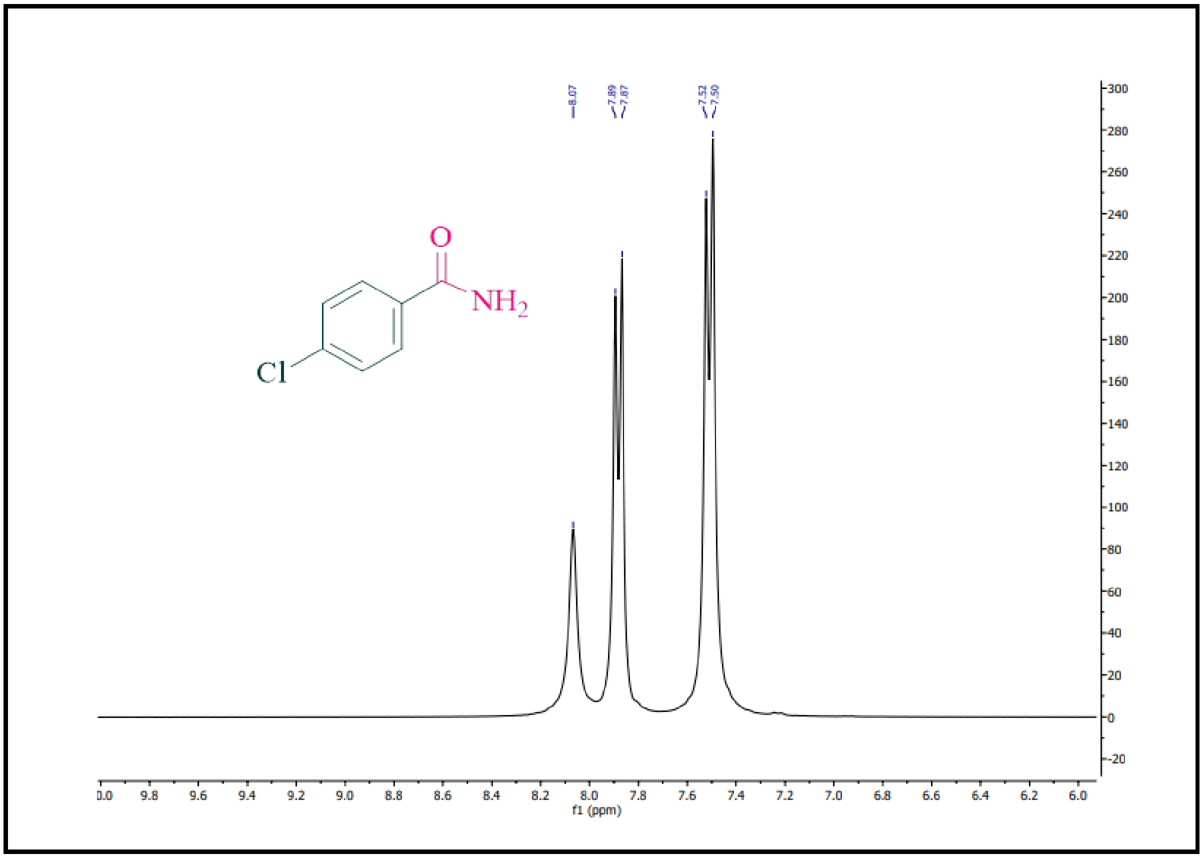
8.07
7.89
7.87
7.52
7.50
O
NH2
Cl
f1 (ppm)

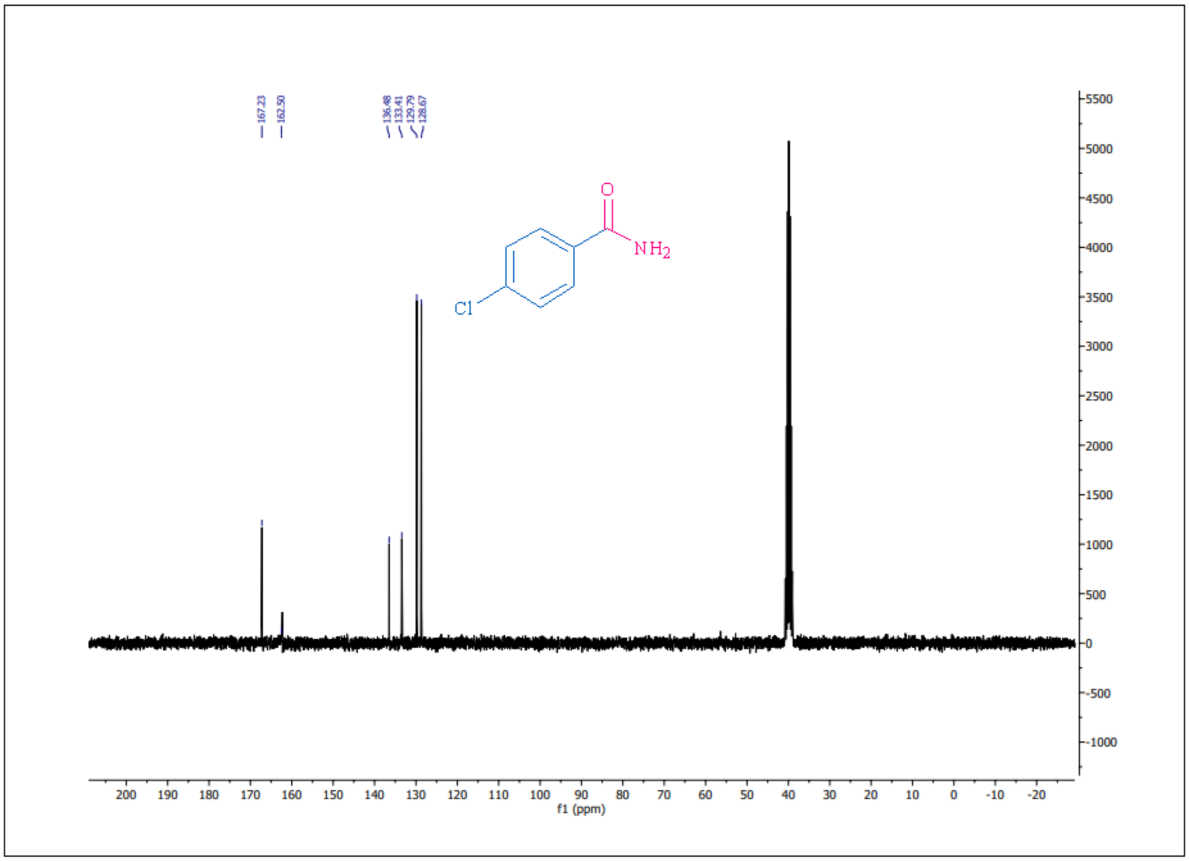

167.23
162.50
136.48
133.41
129.79
128.67
O
NH2
Cl
5500
5000
4500
4000
3500
3000
2500
2000
1500
1000
500
0
-500
-1000
200 190 180 170 160 150 140 130 120 110 100 90 80 70 60 50 40 30 20 10 0 -10 -20
f1 (ppm)

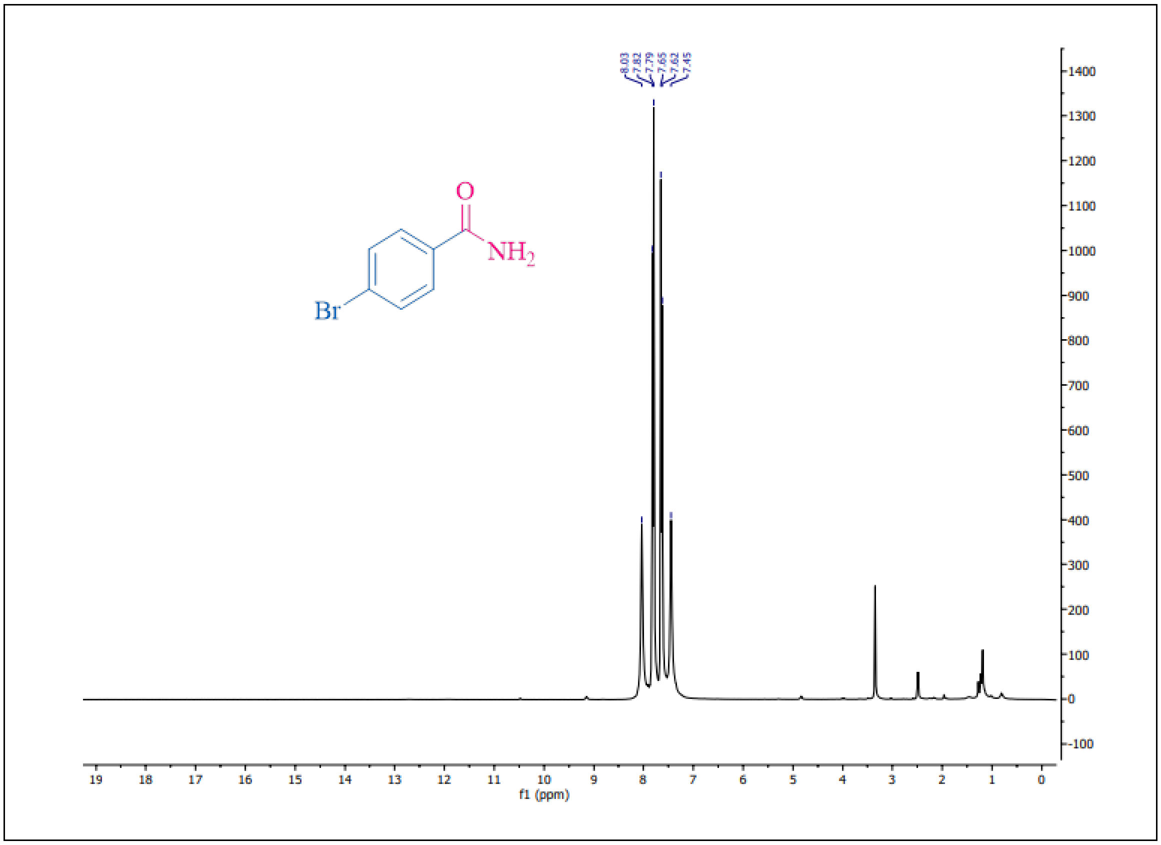
8.03
7.82
7.79
7.65
7.62
7.45
Br
O
NH2
f1 (ppm)

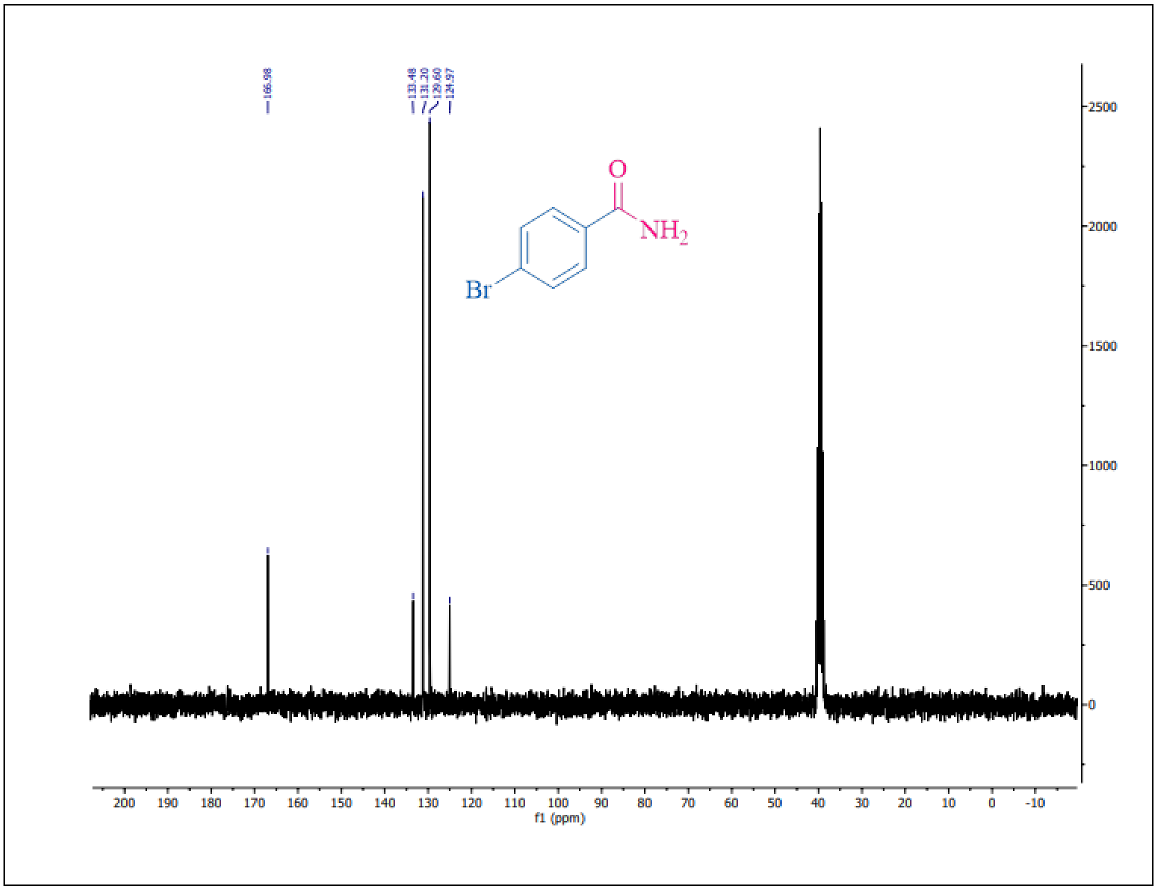

166.98
133.48
131.20
129.60
124.97
O
NH2
Br
2500
2000
1500
1000
500
0
200
190
180
170
160
150
140
130
120
110
100
90
80
70
60
50
40
30
20
10
0
-10
f1 (ppm)

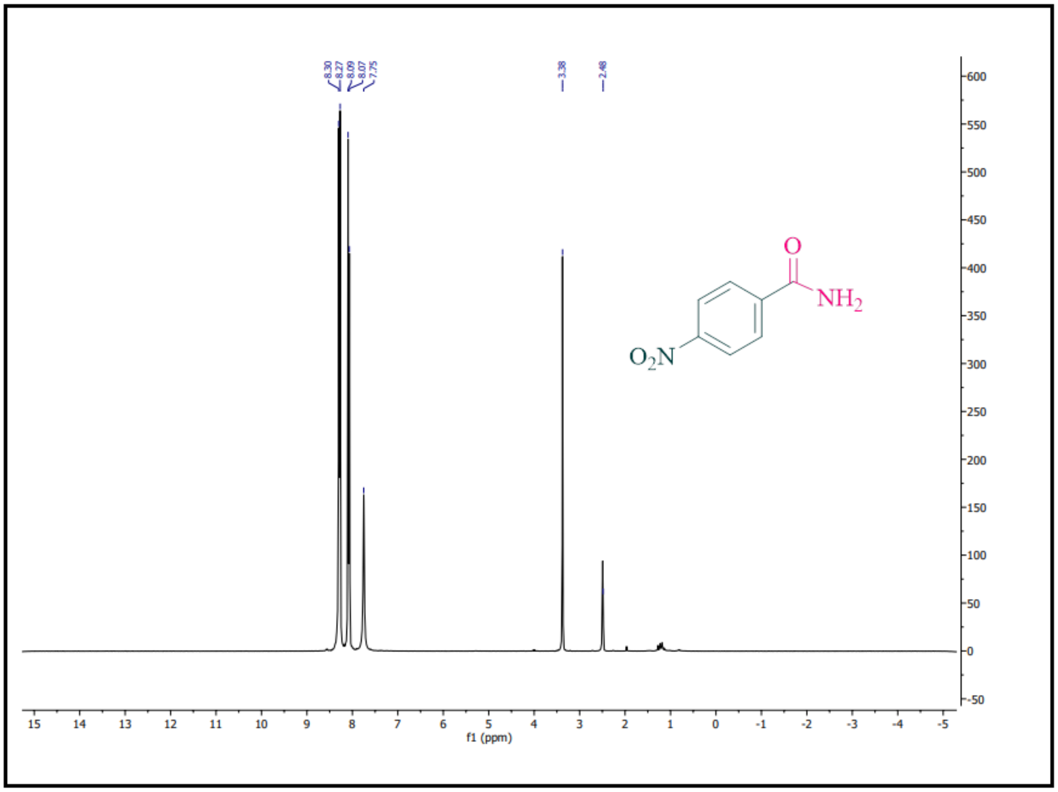
8.30
8.27
8.09
8.07
7.75
3.38
2.48
O
NH2
O2N
600
550
500
450
400
350
300
250
200
150
100
50
0
-50
15
14
13
12
11
10
9
8
7
6
5
4
3
2
1
0
-1
-2
-3
-4
-5
f1 (ppm)

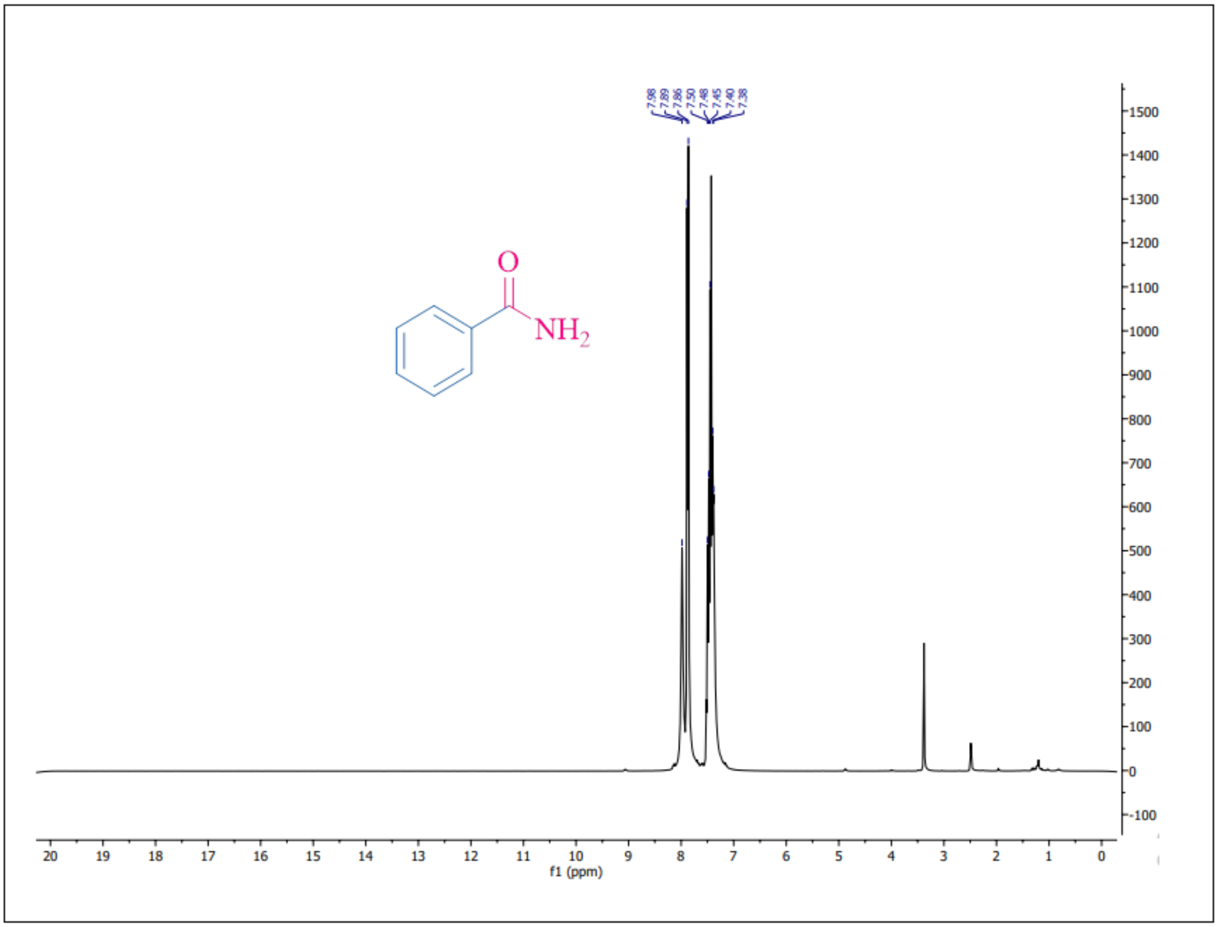

7.98
7.89
7.86
7.50
7.48
7.45
7.40
7.38
O
NH2
1500
1400
1300
1200
1100
1000
900
800
700
600
500
400
300
200
100
0
-100
20 19 18 17 16 15 14 13 12 11 10 9 8 7 6 5 4 3 2 1 0
f1 (ppm)

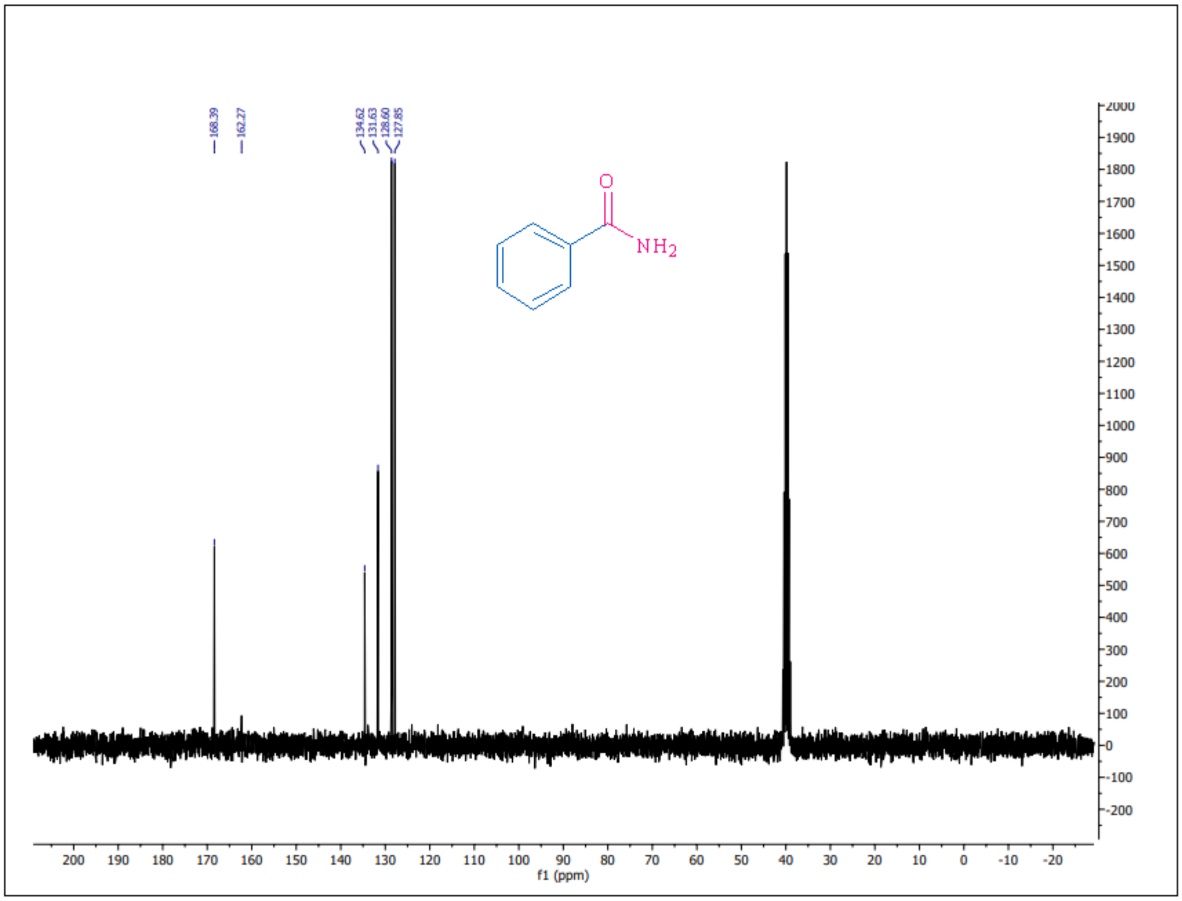

168.39
162.27
134.62
131.63
128.60
127.85
O
NH2
f1 (ppm)

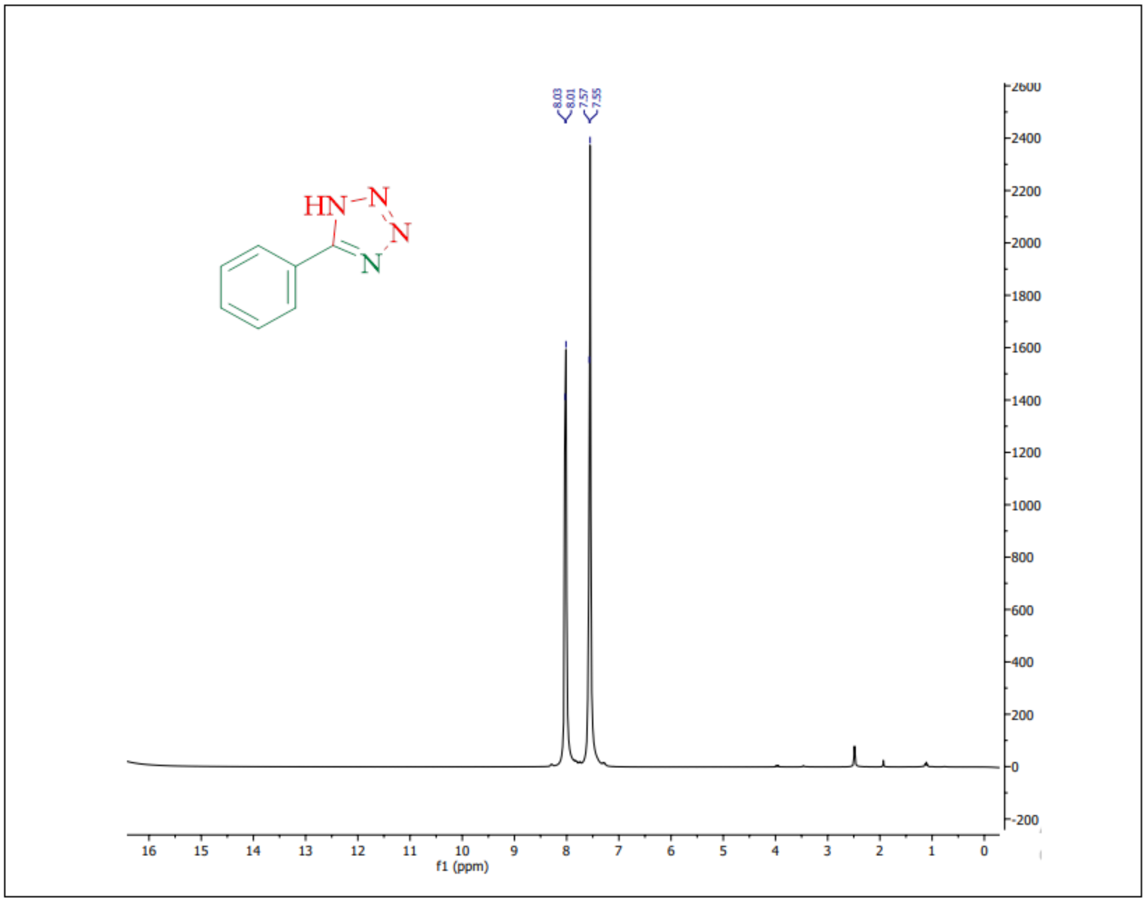
8.03
8.01
7.57
7.55
HN
N
N
N
f1 (ppm)

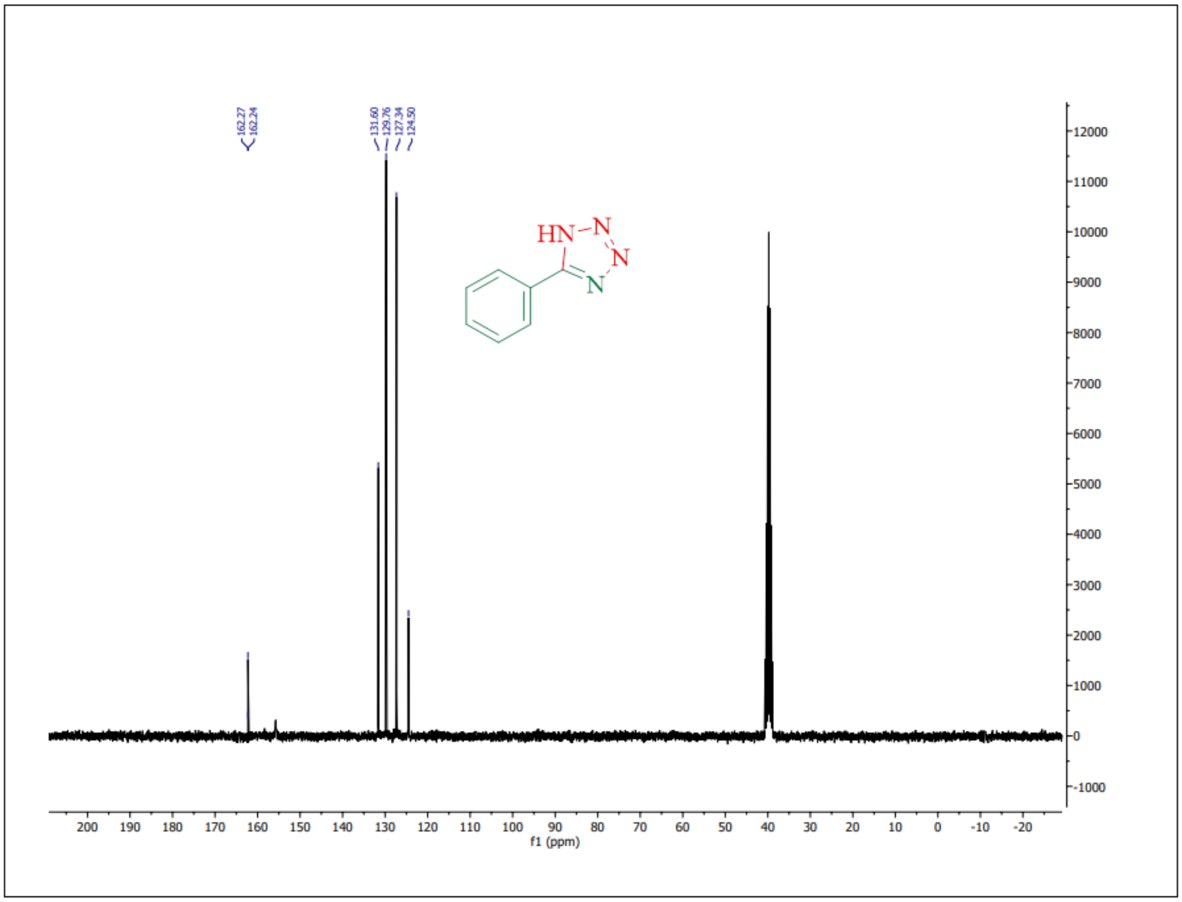
162.27
162.24
131.60
129.76
127.34
124.50
HN
N
N
N
12000
11000
10000
9000
8000
7000
6000
5000
4000
3000
2000
1000
0
-1000
200 190 180 170 160 150 140 130 120 110 100 90 80 70 60 50 40 30 20 10 0 -10 -20
f1 (ppm)

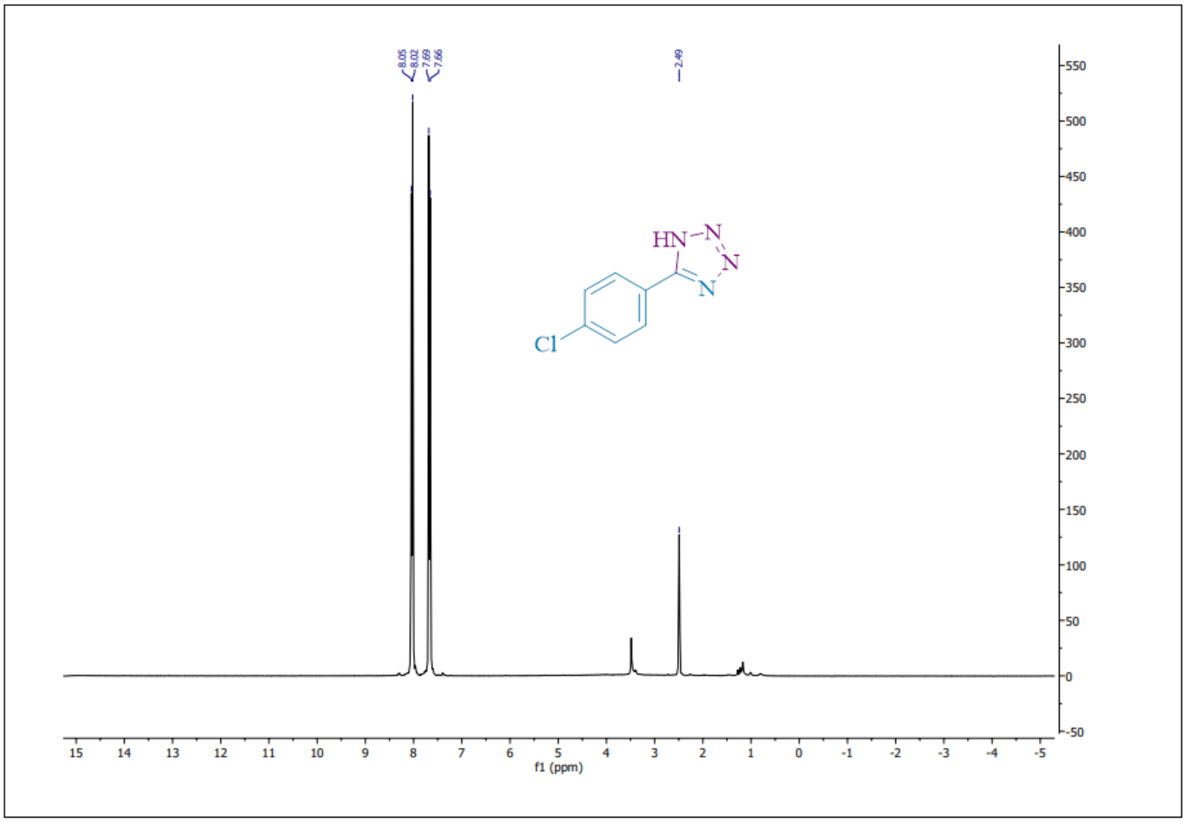
8.05
8.02
7.69
7.66
2.49
HN
N
N
N
Cl
f1 (ppm)

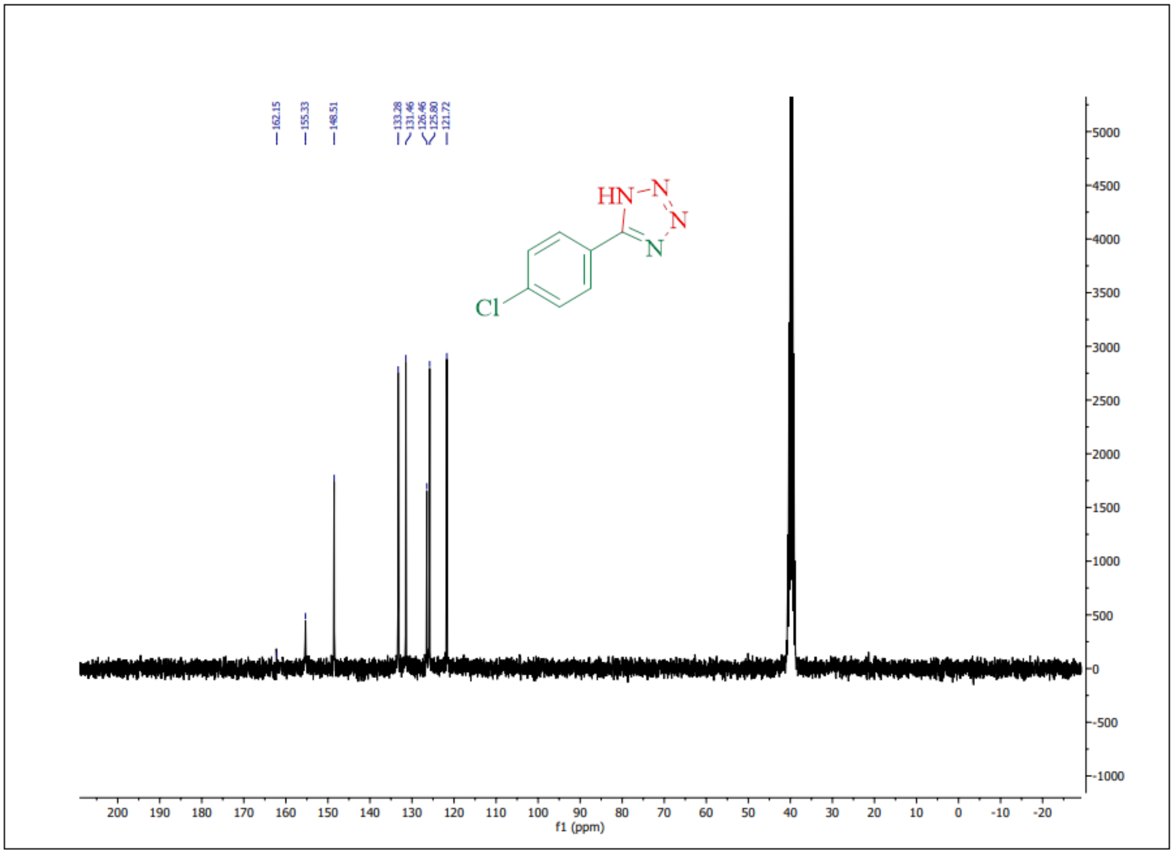

162.15
155.33
148.51
133.28
131.46
126.46
125.80
121.72
HN
N
N
N
Cl
5000
4500
4000
3500
3000
2500
2000
1500
1000
500
0
-500
-1000
200 190 180 170 160 150 140 130 120 110 100 90 80 70 60 50 40 30 20 10 0 -10 -20
f1 (ppm)

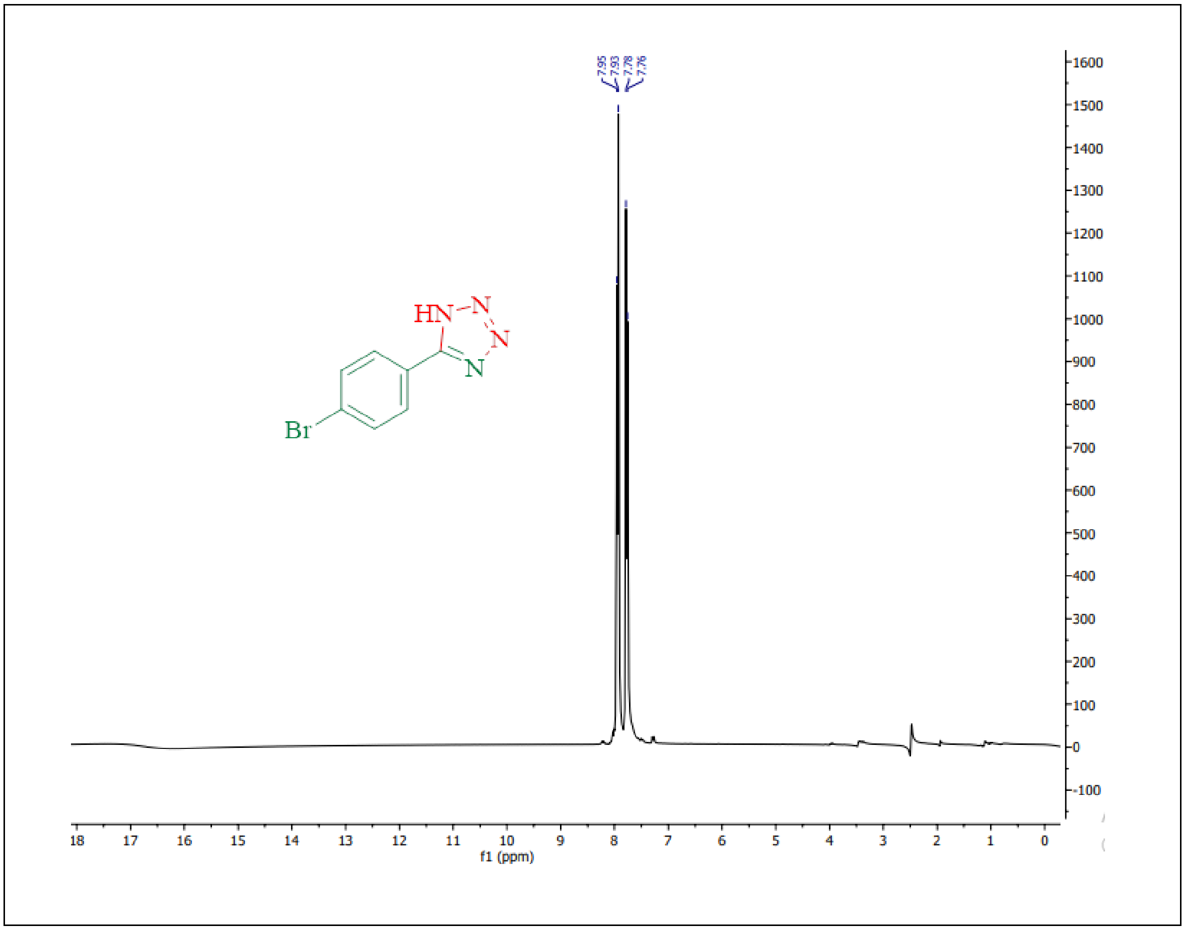
7.95
7.93
7.78
7.76
HN
N
N
N
Br
1600
1500
1400
1300
1200
1100
1000
900
800
700
600
500
400
300
200
100
0
-100
18
17
16
15
14
13
12
11
10
9
8
7
6
5
4
3
2
1
0
f1 (ppm)

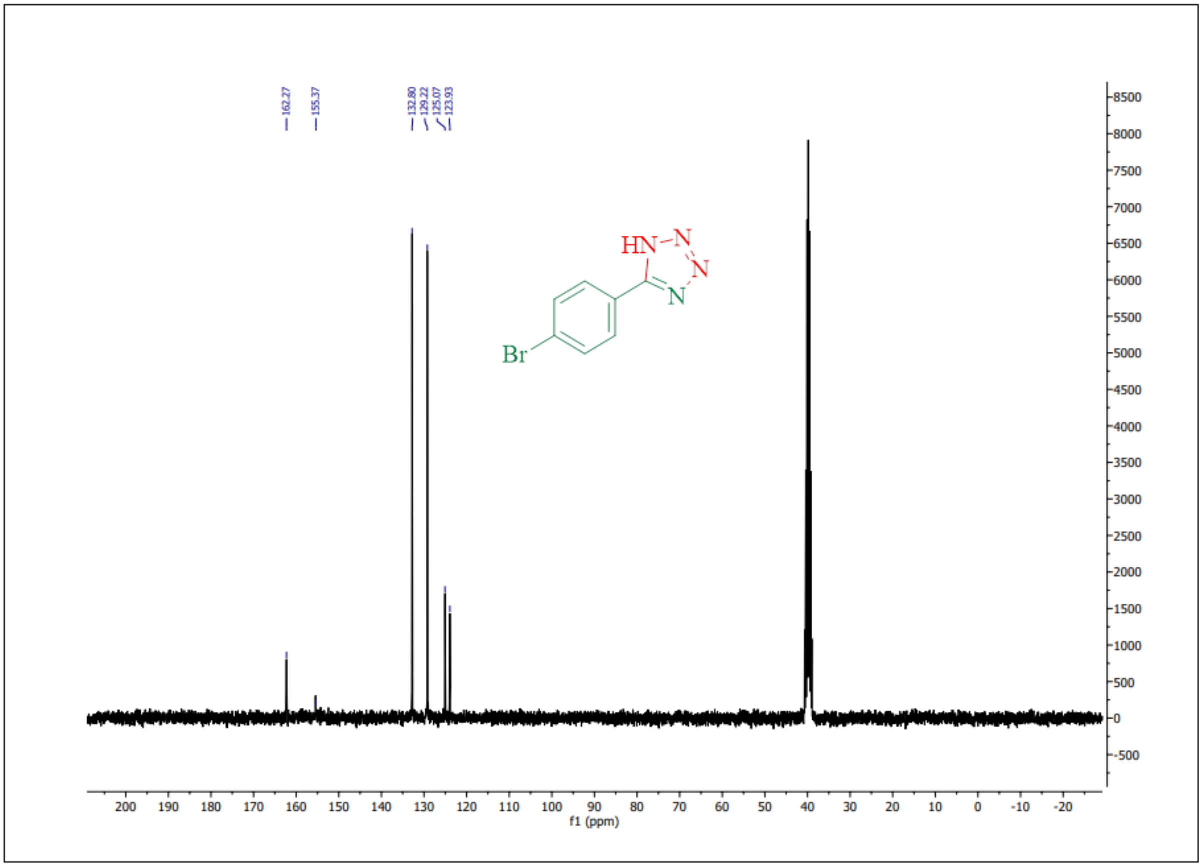
162.27
155.37
132.80
129.22
125.07
123.93
HN
N
N
N
Br
8500
8000
7500
7000
6500
6000
5500
5000
4500
4000
3500
3000
2500
2000
1500
1000
500
0
-500
200 190 180 170 160 150 140 130 120 110 100 90 80 70 60 50 40 30 20 10 0 -10 -20
f1 (ppm)

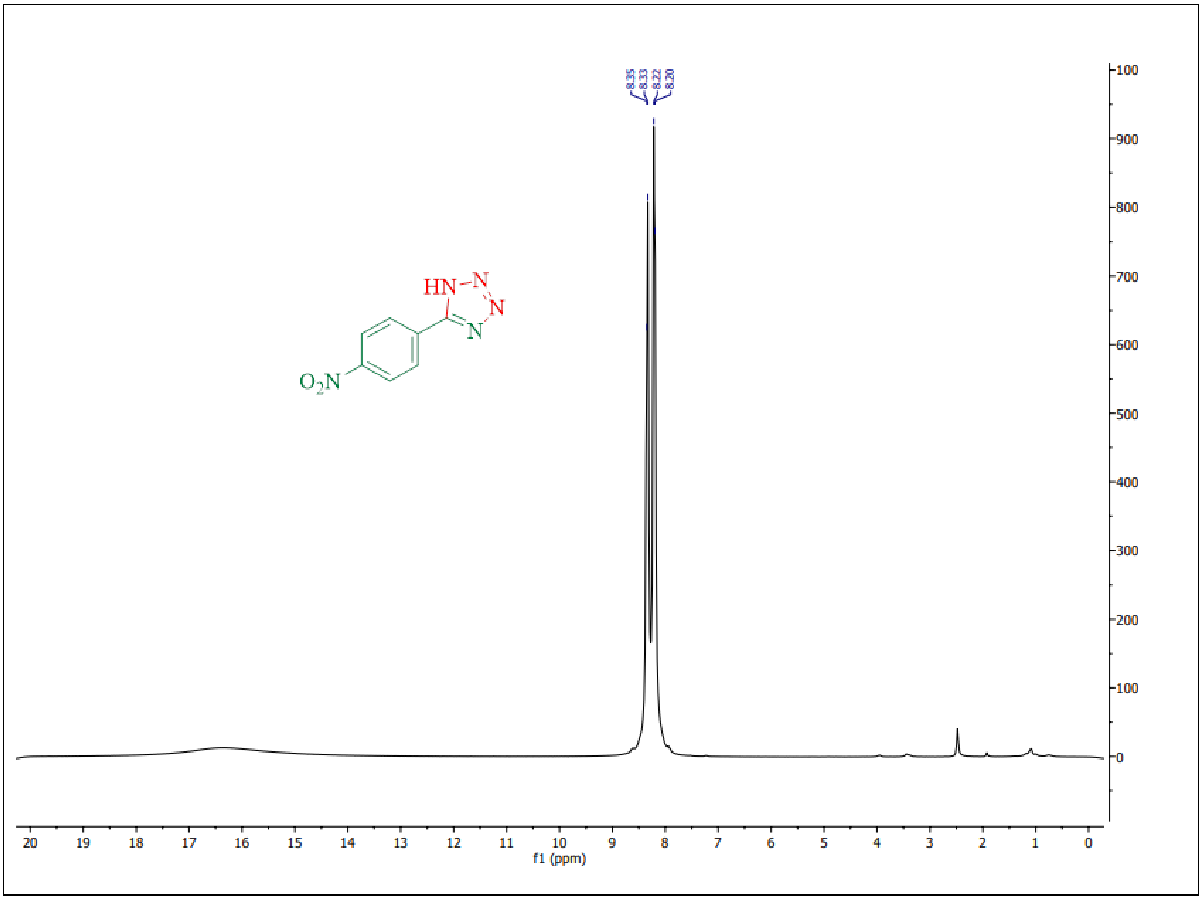

8.35
8.33
8.22
8.20
HN
N
N
N
O_2N
f1 (ppm)

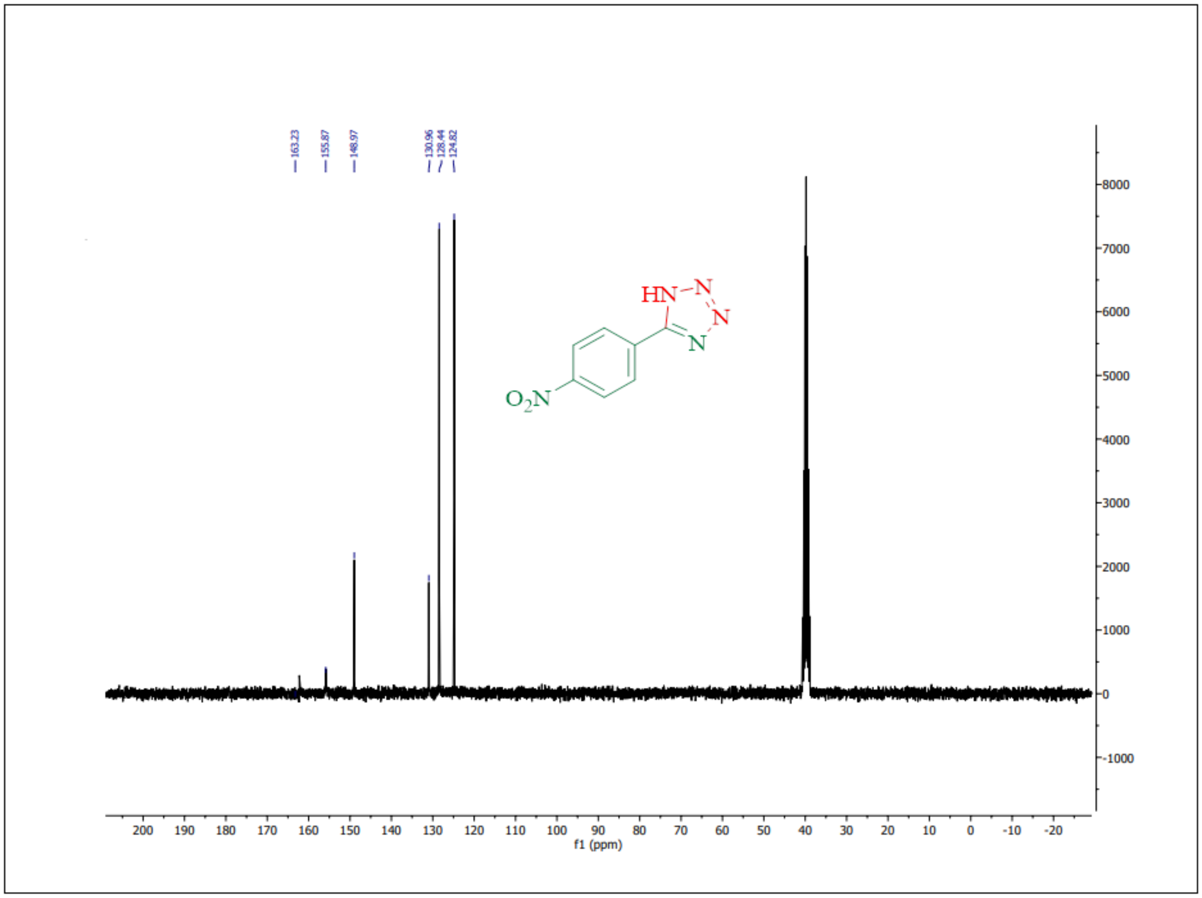
163.23
155.87
148.97
130.96
128.44
124.82
HN
N
N
N
O2N
f1 (ppm)

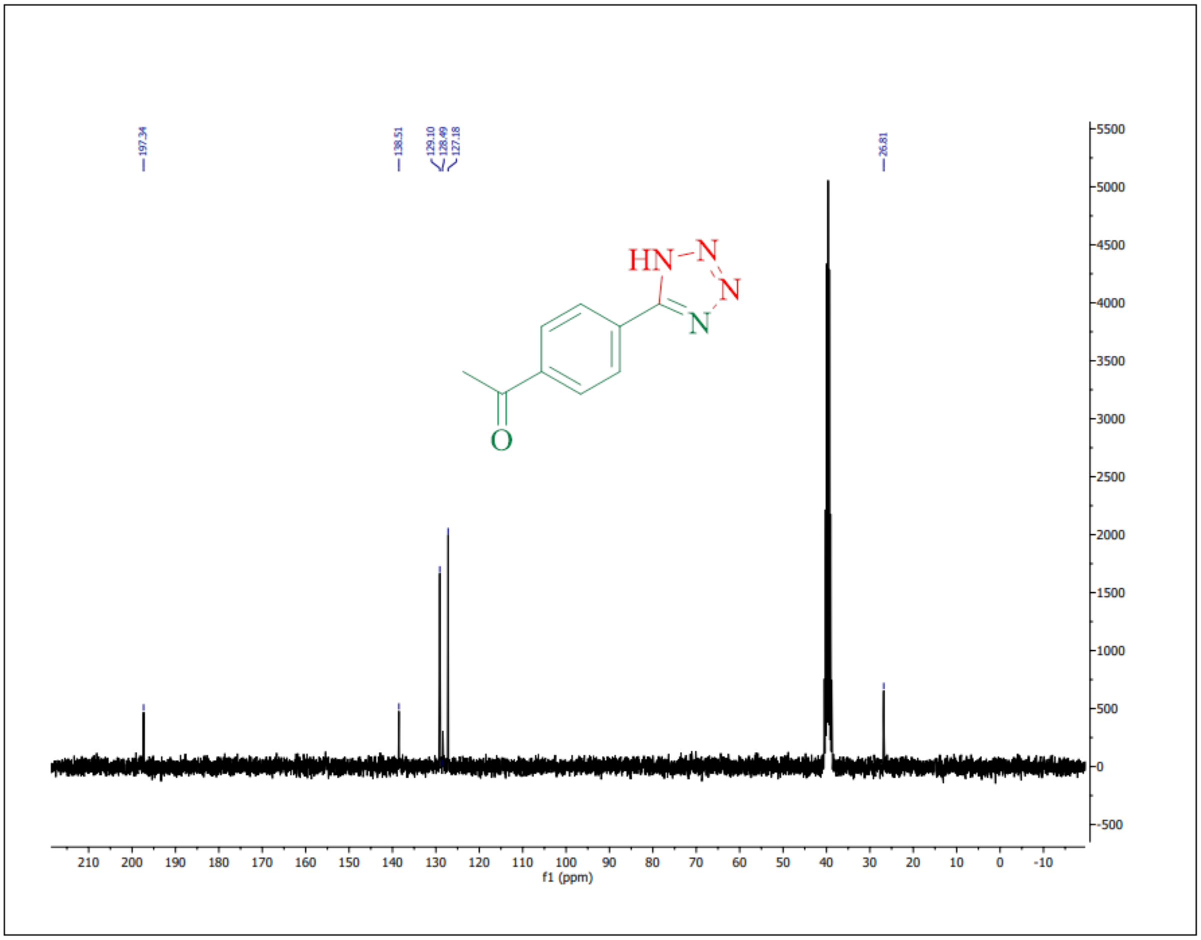
197.34
138.51
129.10
128.49
127.18
26.81
HN
N
N
N
O
5500
5000
4500
4000
3500
3000
2500
2000
1500
1000
500
0
-500
210
200
190
180
170
160
150
140
130
120
110
100
90
80
70
60
50
40
30
20
10
0
-10
f1 (ppm)

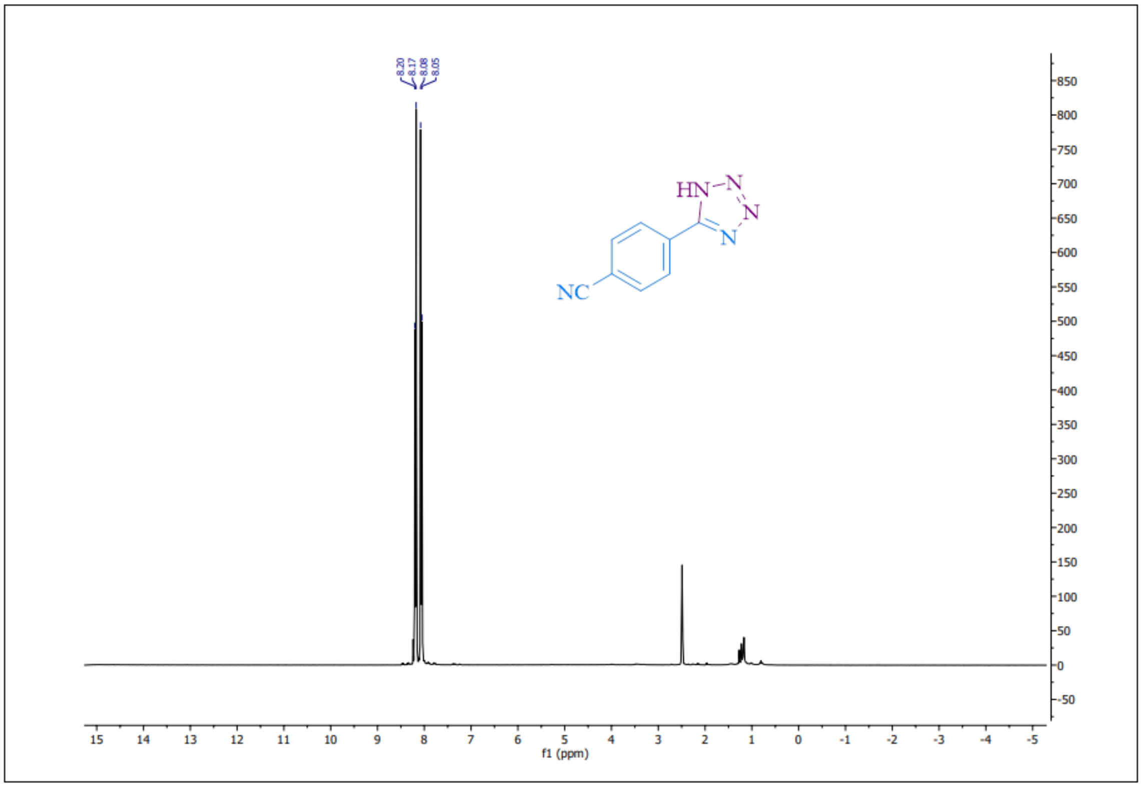

8.20
8.17
8.08
8.05
HN
N
N
N
NC
850
800
750
700
650
600
550
500
450
400
350
300
250
200
150
100
50
0
-50
15
14
13
12
11
10
9
8
7
6
5
4
3
2
1
0
-1
-2
-3
-4
-5
f1 (ppm)

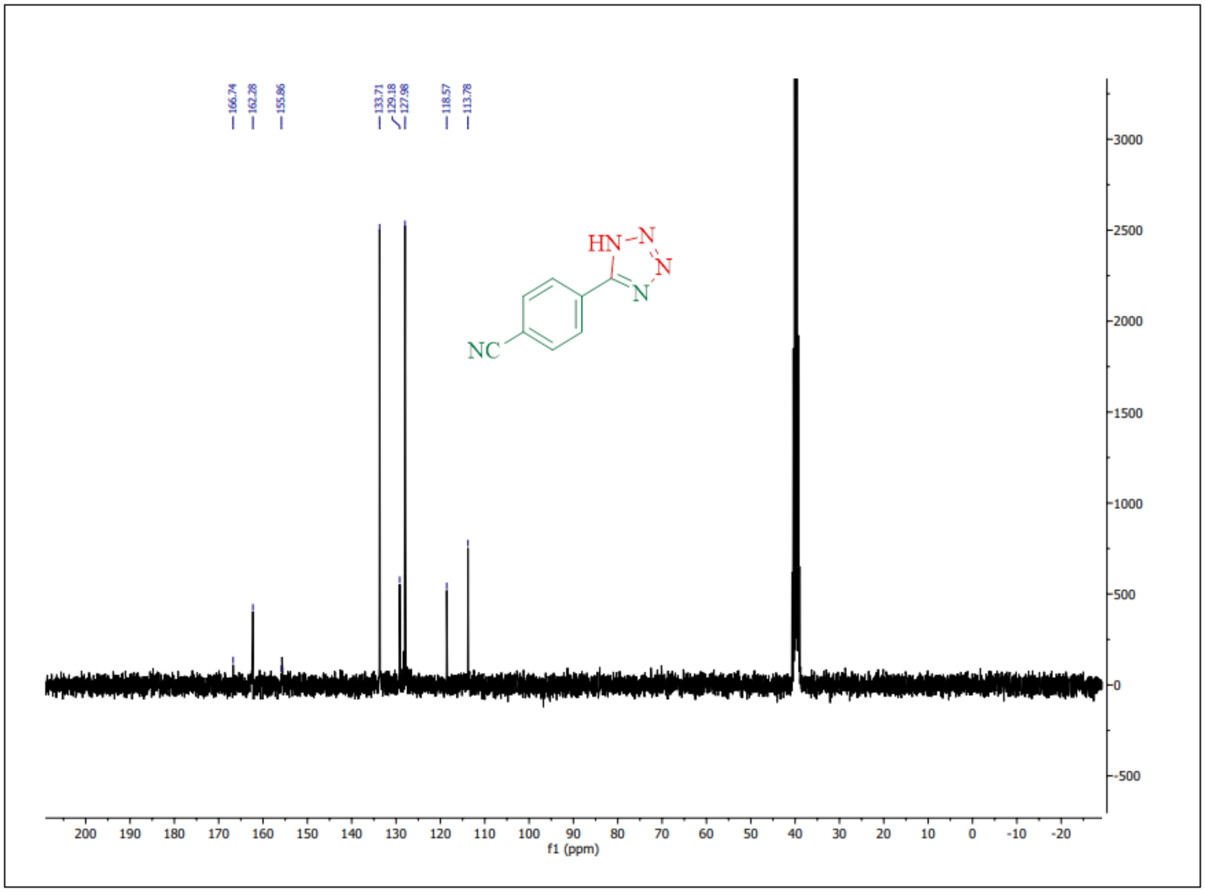

166.74
162.28
155.86
133.71
129.18
127.98
118.57
113.78
HN
N
N
N
NC
3000
2500
2000
1500
1000
500
0
-500
200
190
180
170
160
150
140
130
120
110
100
90
80
70
60
50
40
30
20
10
0
-10
-20
f1 (ppm)

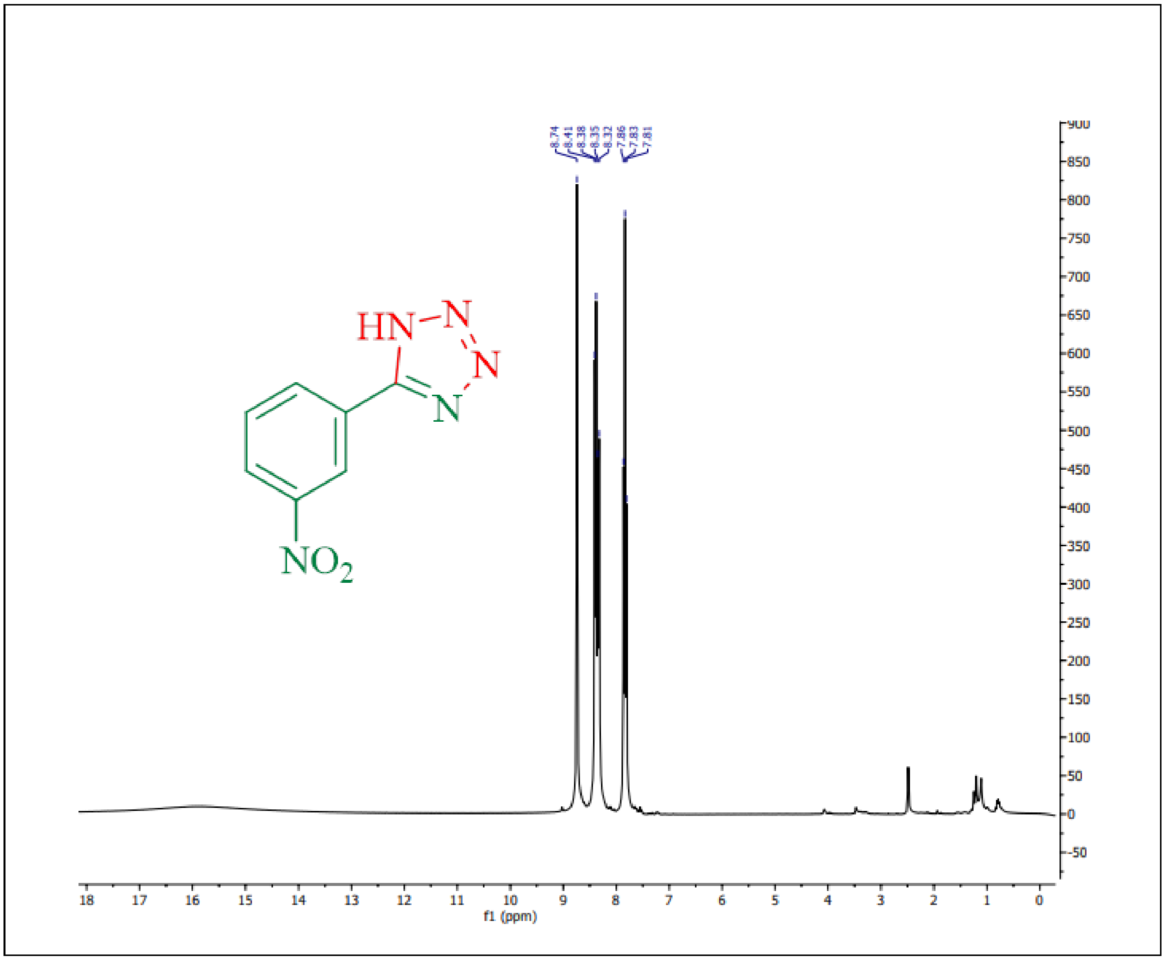
8.74
8.41
8.38
8.35
8.32
7.86
7.83
7.81
HN
N
N
N
NO2
f1 (ppm)

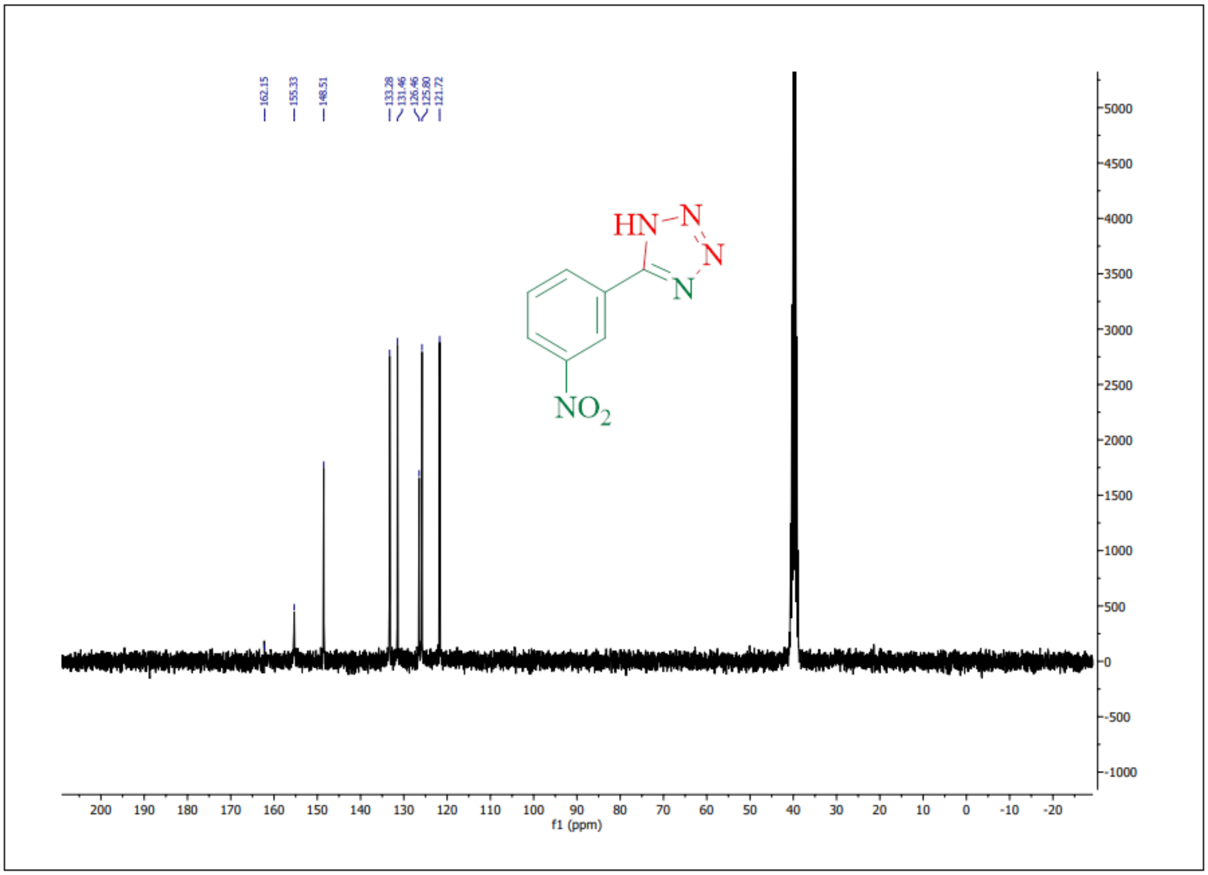
162.15
155.33
148.51
133.28
131.46
126.46
125.80
121.72
HN
N
N
N
NO2
f1 (ppm)

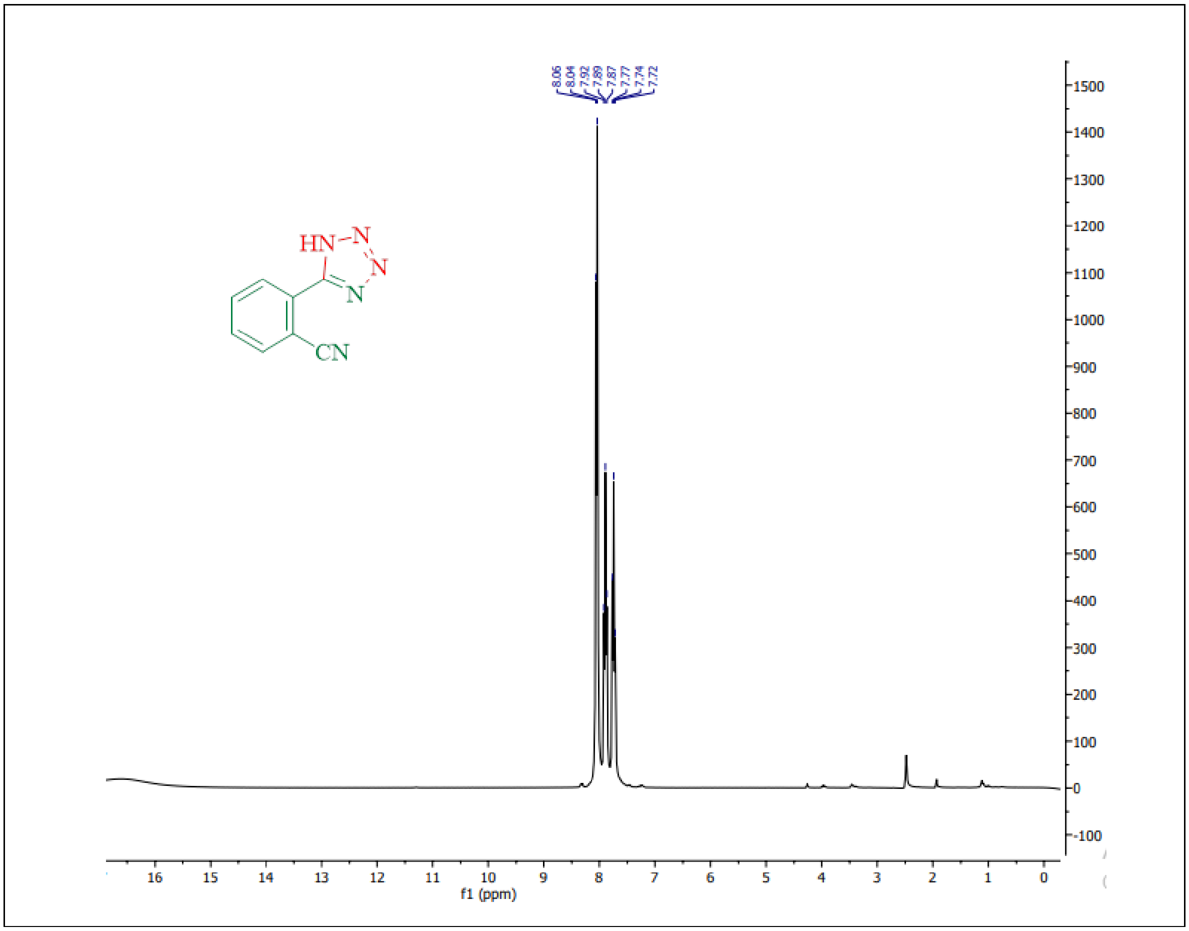
8.06
8.04
7.92
7.89
7.87
7.77
7.74
7.72
HN
N
N
N
CN
1500
1400
1300
1200
1100
1000
900
800
700
600
500
400
300
200
100
0
-100
16 15 14 13 12 11 10 9 8 7 6 5 4 3 2 1 0
f1 (ppm)

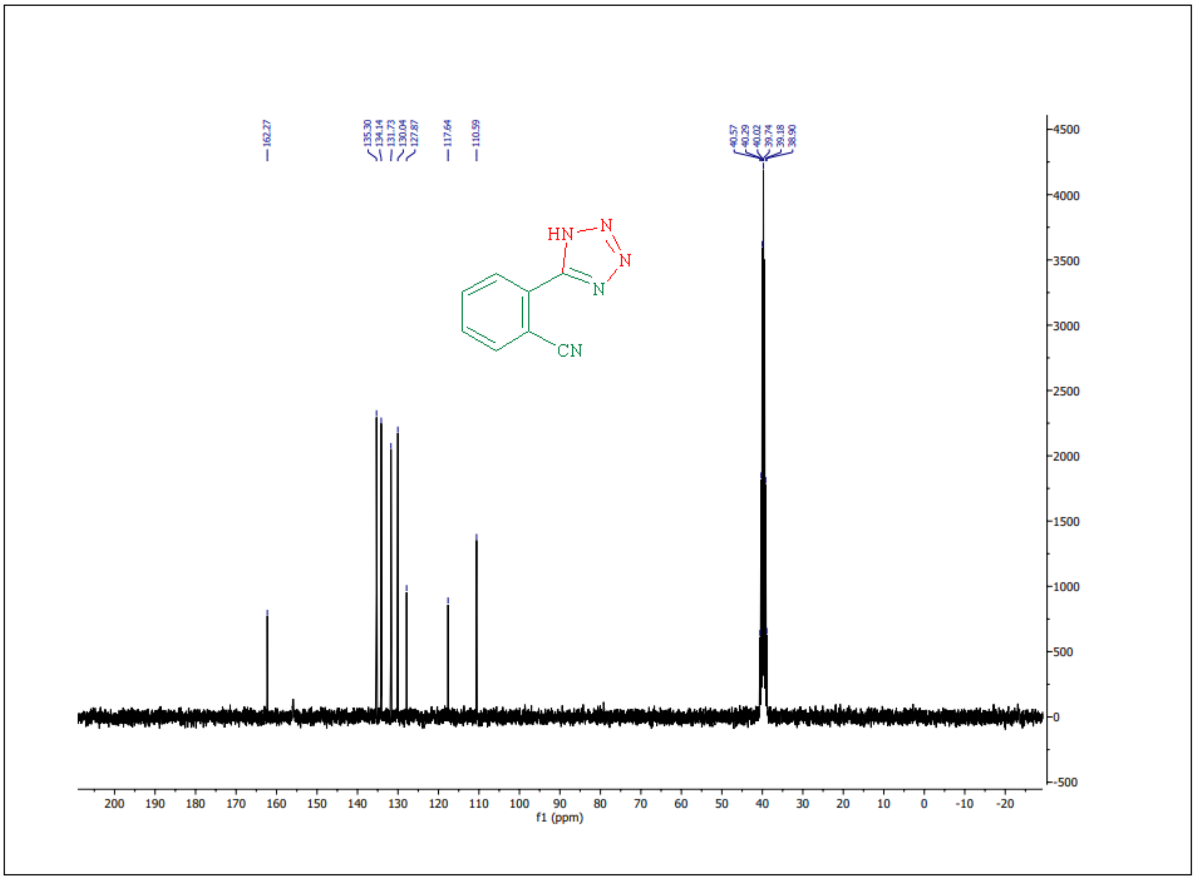
162.27
135.30
134.14
131.73
130.04
127.87
117.64
110.59
40.57
40.29
40.02
39.74
39.18
38.90
HN
N
N
N
CN
f1 (ppm)

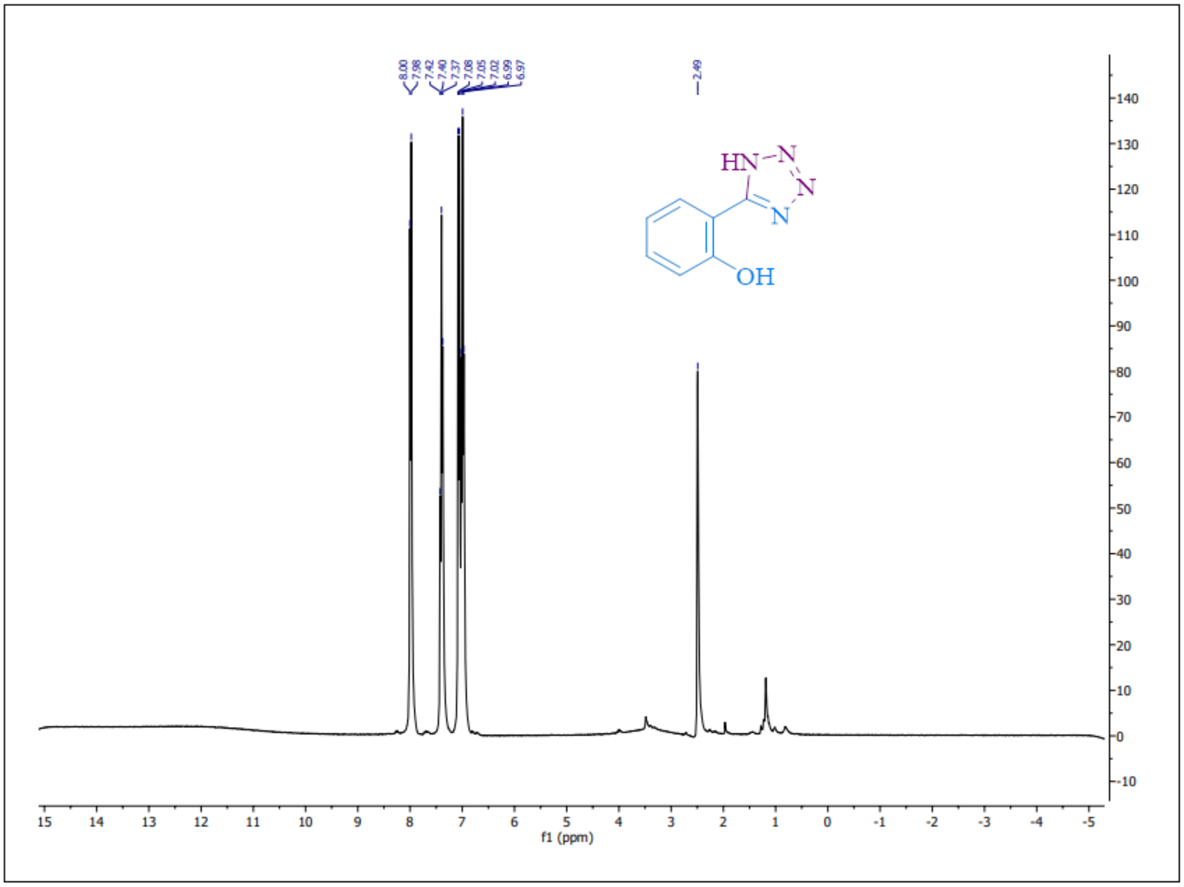
8.00
7.98
7.42
7.40
7.37
7.08
7.05
7.02
6.99
6.97
2.49
HN
N
N
N
OH
f1 (ppm)

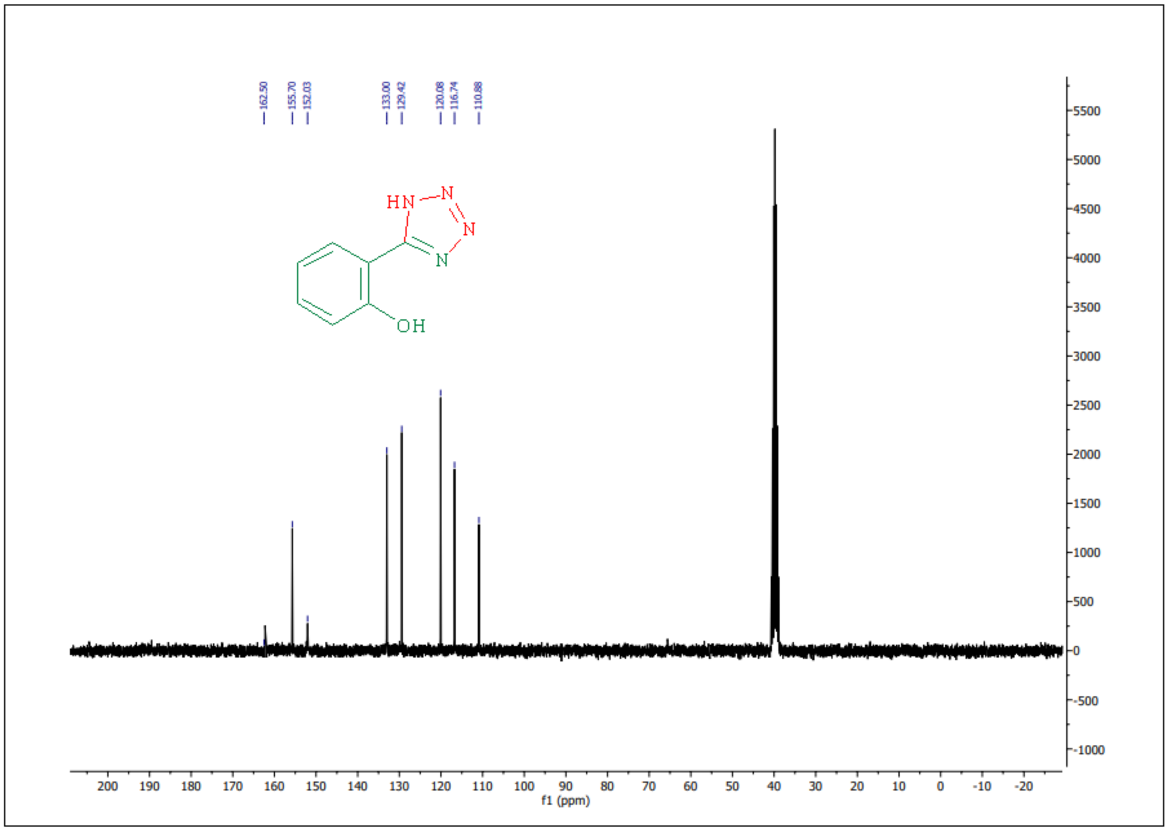

162.50
155.70
152.03
133.00
129.42
120.08
116.74
110.88
HN
N
N
N
OH
f1 (ppm)

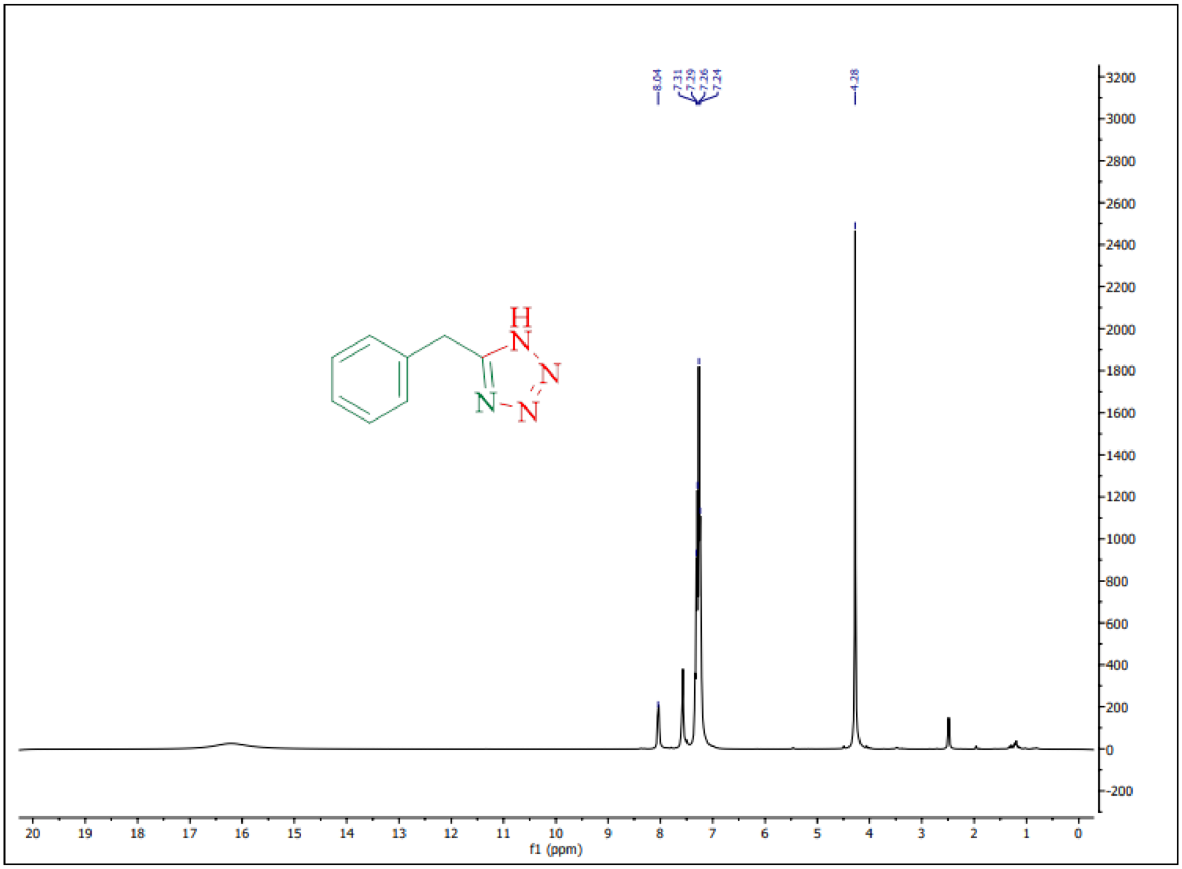
8.04
7.31
7.29
7.26
7.24
4.28
H
N
N
N
N
3200
3000
2800
2600
2400
2200
2000
1800
1600
1400
1200
1000
800
600
400
200
0
-200
20 19 18 17 16 15 14 13 12 11 10 9 8 7 6 5 4 3 2 1 0
f1 (ppm)

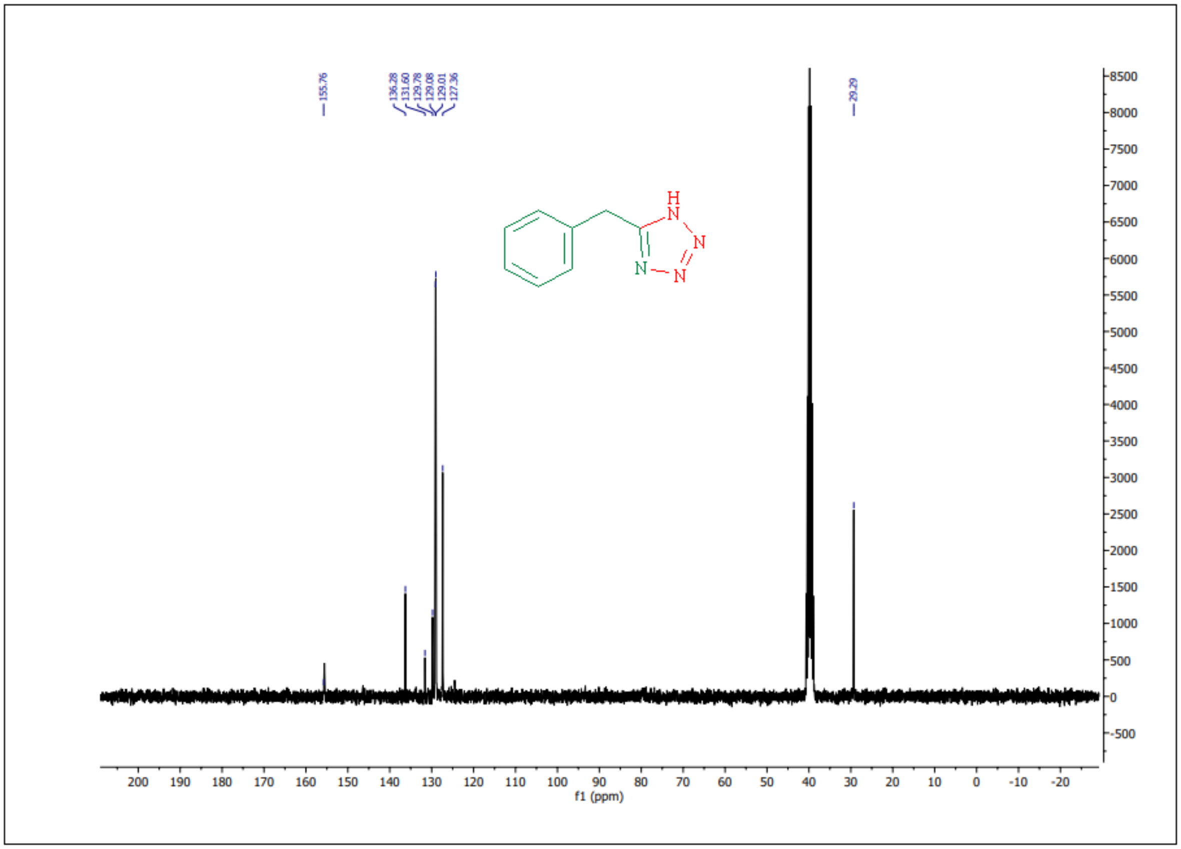

155.76
136.28
131.60
129.78
129.08
129.01
127.36
29.29
H
N
N
N
N
8500
8000
7500
7000
6500
6000
5500
5000
4500
4000
3500
3000
2500
2000
1500
1000
500
0
-500
200 190 180 170 160 150 140 130 120 110 100 90 80 70 60 50 40 30 20 10 0 -10 -20
f1 (ppm)
